# SPION-MSCs enhance therapeutic efficacy in sepsis by regulating MSC-expressed TRAF1-dependent macrophage polarization

**DOI:** 10.1186/s13287-021-02593-2

**Published:** 2021-10-09

**Authors:** Yujun Xu, Xinghan Liu, Yi Li, Huan Dou, Huaping Liang, Yayi Hou

**Affiliations:** 1 The State Key Laboratory of Pharmaceutical Biotechnology, Division of Immunology, Medical School, Nanjing University, 22 Hankou Road, Nanjing, 210093 China; 2 Jiangsu Key Laboratory of Molecular Medicine, Medical School, Nanjing University, Nanjing, 210093 China; 3 State Key Laboratory of Trauma, Burns and Combined Injury, Department of Wound Infection and Drug, Daping Hospital, Army Medical University, Chongqing, China

**Keywords:** Sepsis, MSCs, Macrophages, SPIONs

## Abstract

**Background:**

Sepsis is defined as a life-threatening organ dysfunction caused by a dysregulated host response to infection. The liver has a crucial role in sepsis and is also a target for sepsis-related injury. Macrophage polarization between the M1 and M2 types is involved in the progression and resolution of both inflammation and liver injury. Iron oxide-based synthetic nanoparticles (SPIONs) can be used as antibacterial agents to regulate the inflammatory response. Mesenchymal stromal/stem cells (MSCs) have been widely used in the treatment of autoimmune diseases, sepsis, and other diseases. However, to date, both the effects of SPIONs on MSCs and the fate of SPION-labelled MSCs in sepsis and other diseases are still unclear.

**Methods:**

Mice were subjected to caecal ligation and puncture (CLP) or lipopolysaccharide (LPS) induction to develop sepsis models. The CLP or LPS models were treated with MSCs or SPION-labelled/pretreated MSCs (SPION-MSCs). Bone marrow (BM)-derived macrophages and RAW 264.7 cells were cocultured with MSCs or SPION-MSCs under different conditions. Flow cytometry, transmission electron microscopy, western blotting, quantitative real-time PCR, and immunohistochemical analysis were performed.

**Results:**

We found that SPIONs did not affect the basic characteristics of MSCs. SPIONs promoted the survival of MSCs by upregulating HO-1 expression under inflammatory conditions. SPION-MSCs enhanced the therapeutic efficacy of liver injury in both the CLP- and LPS-induced mouse models of sepsis. Moreover, the protective effect of SPION-MSCs against sepsis-induced liver injury was related to macrophages. Systemic depletion of macrophages reduced the efficacy of SPION-MSC therapy. Furthermore, SPION-MSCs promoted macrophages to polarize towards the M2 phenotype under sepsis-induced liver injury in mice. The enhanced polarization towards M2 macrophages was attributed to their phagocytosis of SPION-MSCs. SPION-MSC-expressed TRAF1 was critical for promotion of macrophage polarization and alleviation of sepsis in mice.

**Conclusion:**

MSCs labelled/pretreated with SPIONs may be a novel therapeutic strategy to prevent or treat sepsis and sepsis-induced liver injury.

**Highlights:**

1. SPIONs enhance the viability of MSCs by promoting HO-1 expression.
2. SPION-labelled/pretreated MSCs effectively improve sepsis by regulating macrophage polarization to M2 macrophages.
3. SPION-labelled/pretreated MSCs regulate macrophage polarization in a manner dependent on MSC-expressed TRAF1 protein.

**Supplementary Information:**

The online version contains supplementary material available at 10.1186/s13287-021-02593-2.

## Background

Sepsis was previously considered a fatal organ dysfunction caused by an imbalance in the host reaction imbalance to infection. The updated definition of sepsis involves the imbalance of the body's response to infection leading to life-threatening organ dysfunction, which is reflected in physiological and biochemical abnormalities at the cellular level. Despite continuous progress in the development of therapeutic strategies, sepsis remains the leading cause of death in the critically ill patient population due to uncontrolled inflammation together with immunosuppression [1, 2]. The liver has crucial roles in sepsis, as it represents an important line of defence against microorganisms, as well as a frequent target of dysregulated inflammation. Sepsis-induced liver injury has a substantial effect on outcome in sepsis, mostly due to altered bacterial or lipopolysaccharide (LPS) clearance and the increased release of proinflammatory cytokines that promote dysfunction of distal organs. Thus, how to regulate immune cells and proinflammatory cytokines to treat sepsis and sepsis-induced liver injury has become a research focus.

Mesenchymal stromal/stem cells (MSCs), nonhaematopoietic adult stem cell-like cells, show multidirectional differentiation and are involved in haematopoietic support and tissue repair. MSCs also modulate immune and inflammatory properties via soluble factors, exosomes, and cell–cell interactions [3, 4]. MSCs have been used in animal experiments of various immune diseases and diseases related to tissue damage and immune diseases [5–7]. Growing preclinical data suggest that MSCs can directly modify the pathophysiology and underlying injury-related mechanisms in sepsis through immunomodulatory, antibacterial, anti-inflammatory, antioxidative, antiapoptotic, and reparative properties. Condor et al. [8] recently analysed the efficacy of MSCs derived from human Wharton’s jelly (WJ-MSCs) in rat models of caecal ligation and puncture (CLP)-induced sepsis. In the animals receiving WJ-MSCs 6 h after CLP, the 5-day survival was significantly increased, and both liver and kidney functions were improved. Wang et al. [9] also reported the ability of murine derma-derived MSCs (DMCs) to attenuate CLP-induced sepsis.

Iron oxide nanoparticles have been used as carriers for the diagnosis and treatment of sepsis [10]. Some nanoiron oxides have also been used as antibacterial agents to regulate the inflammatory response and biomarkers of sepsis [11]. Treatment of an LPS-induced inflammatory mouse model with drugs loaded in albumin-modified iron oxide nanoparticles reduced the infiltration of neutrophils and monocytes into inflammatory sites. We previously found that iron oxide-based synthetic nanoparticles (SPIONs) activated macrophage autophagy through the Cav1-Notch1/HES1 signalling pathway, which in turn promoted macrophages to produce IL-10 and alleviated liver injury in LPS-induced septic mice [12]. In addition, we found that SPIONs reduced the percentage of myeloid-derived suppressor cells in the spleen of LPS-induced septic mice [13].

Macrophages are important participants in the inflammatory response and play an important role in inflammatory diseases. Currently, macrophages can be categorized into two opposite types: the M1 phenotype and the M2 phenotype. M1 macrophages are proinflammatory and produce proinflammatory cytokines. M2 macrophages are anti-inflammatory and immunoregulatory and produce anti-inflammatory cytokines. In the early stage of sepsis, to eliminate invading bacteria, macrophages are activated to become the M1 type, which produces a large number of proinflammatory factors, such as tumour necrosis factor (TNF)-α, interleukin (IL)-6, IL-1β, type I interferon, and chemokines. Excessive inflammation leads to lymphocyte apoptosis, and infiltration of macrophages into tissues causes tissue damage and organ failure. Therefore, inhibiting excessive M1-type macrophages and promoting M2-type macrophages in sepsis will help improve the disease. Many studies have shown that MSCs can regulate the polarization of macrophages from the inflammatory M1 to the anti-inflammatory M2 type in vitro and in vivo.

In recent years, various types of nanoparticles have been designed to mark and track MSCs and used as gene/drug delivery carriers to regulate the biological behaviour and function of MSCs. It was reported that MSCs labelled with iron oxide particles reach the liver and spleen immediately after being injected into the body, and the clearance half-life of MSCs labelled with iron oxide in the liver is 4.6 days [14]. It was also reported that SPIONs successfully labelled MSCs and did not affect cell viability or differentiation potential [15]. Moreover, SPION-labelled/pretreated MSCs enhanced the expression of transferrin receptors. The interaction between bone marrow-derived MSCs labelled with silicon nanoparticles (PMSNs) and Ly6C inflammatory monocytes promoted the expression of CCR2 on monocytes, which improved the curative effect of MSCs on myocardial infarction [16, 17]. These data indicate that optimizing and improving MSCs cultured in vitro may restore the balance between proinflammatory and anti-inflammatory systems in patients with sepsis as a new treatment strategy for sepsis.

We recently revealed that the TLR3 ligand poly(I:C) promoted MSCs to produce more PGE2, COX-2, IDO, and IL-8 and enhanced the immunosuppressive function of MSCs [18–20]. MSCs pretreated with IL-1β also alleviated experimental colitis in mice [21], and midkine (MK) promoted the expansion of MSCs in vitro [22]. Of note, SPIONs are characterized by low toxicity and good sensitivity [23–25] and are commonly used as stem cell tracers [26–28] and for clinical magnetic resonance imaging [29–31]. However, to date, the detailed effects of SPIONs on MSCs and the fate of SPION-labelled MSCs in sepsis and other diseases are unclear.

Here, we found that SPIONs did not affect the basic characteristics of MSCs but promoted the survival of MSCs by upregulating HO-1 expression. SPION-labelled/pretreated MSCs (SPION-MSCs) significantly improved CLP- or LPS-induced sepsis and liver injury in mice and enhanced macrophage polarization to the M2 type. Mechanistically, TRAF1 expression in MSCs pretreated with SPIONs was critical for polarization of macrophages to the M2 type and alleviation of sepsis in mice.

## Methods

### Reagents and antibodies

Recombinant human TNF-α protein was purchased from R&D (Boston, MO, USA). Clodronate liposome was purchased from Liposoma B.V (Vrije University, Netherlands). Rhodamine B and LPS were purchased from Sigma (St. Louis, MO, USA). Anti-Arg1, anti-IL-10, anti-iNOS, anti-TRAF1, anti-p-p65, anti-p65, anti-Cox2, anti-Ho-1, anti-Bcl2, anti-Bax, and anti-GAPDH antibodies were purchased from Cell Signaling Technology (Boston, MO, USA). SPIONs were provided by Professor Ning Gu’s team at Southeast University, Nanjing, PR China.

### Mice and septic models

C57BL/6 mice were purchased from the Model Animal Research Center of Nanjing University. Mice were raised in the General Clinical Research Center of Nanjing First Hospital of Nanjing Medical University. All the mice used in this study were housed with a normal 12 h/12 h light/dark cycle. Mice had access to food and water ad libitum. The CLP-induced septic model was induced by an operation in male C57BL/6 mice (25–30 g). In the sham-operated mice, we exteriorized the caecum but neither ligated nor punctured it. After surgery, we resuscitated the mice by injecting prewarmed normal saline (37 °C; 1 mL per mouse, subcutaneously). The CLP-operated mice were randomly assigned to receive injections of phosphate-buffered saline (PBS), SPION-MSCs (1 × 10^6^ cells/mouse), or MSCs (1 × 10^6^ cells/mouse) by the tail vein 4 h after the operation. Blood, peritoneal lavage fluid, liver, lung, and kidney tissues were collected for subsequent determination 2 days or 7 days after the CLP operation.

C57BL/6 mice were intraperitoneally administered LPS (5 mg/kg) for 4 h followed by treatment with PBS, SPION-MSCs (1 × 10^6^ cells/mouse), or MSCs (1 × 10^6^ cells/mouse) through the vena caudalis. After 2 days or 7 days, blood, peritoneal lavage fluid, liver, lung, and kidney tissues were collected for subsequent determination.

### Elimination of mouse macrophages

For depletion of macrophages, C57BL/6 mice were intraperitoneally injected with 200 μL of clophosome-A clodronate liposomes (Clo-Lip) into the abdominal cavity, followed by CLP. The CLP-operated mice were randomly assigned to receive injections of PBS or SPION-MSCs (1 × 10^6^ cells/mouse) into the tail vein 4 h after the operation, and the mice were administered 200 μL of Clo-Lip after 24 h. After 1 day, the mice were euthanized. The liver, blood, and spleen were collected for flow cytometry analysis. The liver was also collected for immunofluorescence detection and HE staining, and plasma was detected by enzyme-linked immunosorbent assay (ELISA) and biochemical analysis.

### Culture and labelling/pretreating of MSCs

The umbilical cord-derived MSCs were obtained from the Stem Cell Center of Jiangsu Province, China. The detailed purification and identification procedures were described previously [32, 33]. MSCs were cultured in Dulbecco's modified Eagle's medium (DMEM) with low glucose containing 10% foetal bovine serum (FBS; Gibco, Carlsbad, CA). For labelling/pretreating MSCs with SPIONs, MSCs were engulfed in SPIONs to obtain SPION-bearing MSCs (SPION-MSCs).

### Cell culture

The mouse macrophage line RAW 264.7 and the mouse fibroblast line L929 were obtained from the Shanghai Institute of Cell Biology (Shanghai, China) and cultured in DMEM (Gibco, Carlsbad, CA) with 10% FBS.

Bone marrow-derived macrophages (BMDMs) were obtained from 8-week-old female C57BL/6 mice. Cells were cultured in RPMI 1640 medium (Gibco) supplemented with 50 ng/mL M-CSF (Peprotech, Rocky Hill, USA), antibiotics, and 10% FBS. The medium was replaced on Day 3, and the cells were collected and used for experiments on Day 5. All cells were incubated at 37 °C in a humidified environment with 5% CO_2_.

### Transmission electron microscopy

Liver tissues were processed for ultrastructural analysis by transmission electron microscopy (TEM). Liver tissues were fixed in 2.5% glutaraldehyde in 0.1 M phosphate buffer (pH = 7.4) at room temperature for 30 min and further fixed overnight in the refrigerator. The samples were rinsed in 0.1 M phosphate buffer and centrifuged prior to postfixation using 2% osmium tetroxide in 0.1 M phosphate buffer (pH = 7.4) at 4 °C for 2 h. Following postfixation, the cells were dehydrated stepwise in ethanol followed by acetone and LX-112 infiltration and finally embedded in LX-112. Ultrathin sections (approximately 50–60 nm) were prepared using a Leica EM UC6, contrasted with uranyl acetate followed by lead citrate, and examined under a Hitachi HT 7700 electron microscope (Hitachi High-Technologies). Digital images were acquired using a 2kx2k Veleta CCD camera (Olympus Soft Imaging Solutions).

### Labelling SPIONs with rhodamine B

Twenty milligrams of SPIONs, 0.2 mg of rhodamine B (RhB), and 5 mL of DMSO were dissolved in a 10 mL conical flask and stirred overnight at room temperature. The solution was diluted with 20 mL of deionized water and transferred to a dialysis bag (3.5 kDa MWCO). Then, the solution was dialyzed with 2 L of deionized water for 2 days to remove unlabelled fluorescent dye and freeze-dried into powder. The powder was evenly mixed using ultrapure water and filtered through a 0.22 µm filter. SPIONs labelled with rhodamine B (SPIONs-RhB) were obtained.

MSCs were inoculated and grown to the logarithmic growth phase in a 6-well plate, cultured with SPION-RhB, and observed under an inverted fluorescence microscope according to the experimental time. After successful labelling, MSCs showed orange fluorescence for the subsequent tests.

### MSCs or L929 cells cocultured with macrophages

#### Indirect coculture

MSCs were seeded in standard 6-well plates or in the upper compartment of Transwells (0.4-μm pore size, Costar, Corning, Tewksbury, MA, http://www.corning.com) and then treated with 200 µg/mL SPIONs or vehicle for 12 h, followed by two washes with PBS. RAW 264.7 cells or BMDMs were collected and cocultured with MSCs in the lower compartment of Transwells. RAW 264.7 cells were then coincubated in the presence of LPS (100 ng/mL) for 24 h. The supernatants were collected for ELISAs, and cells were collected for flow cytometry analysis or protein extraction.

#### Direct coculture

MSCs were seeded in standard 6-well plates and then treated with 200 µg/mL SPION-RhB or DM-Dil for 12 h. L929 cells were seeded in standard 6-well plates and then treated with 200 µg/mL SPION-RhB for 12 h, followed by two washes with PBS. RAW 264.7 cells were collected and cocultured with MSCs (MSCs:RAW 264.7 = 1:4) or L929 cells (L929 cells:RAW 264.7 = 1:4). RAW 264.7 cells were then coincubated in the presence of LPS (100 ng/mL) for 24 h. The supernatants were collected for ELISAs, and cells were collected for flow cytometry.

### Fluorescent imaging of phagocytosis of RAW 264.7 cells to MSCs

RAW 264.7 cells were cultured at 37 °C on standard 6-well plates for 12 h, 24 h, and 48 h in the presence of SPION-RHB-labelled MSCs at a 1:4 ratio (MSC:RAW 264.7) in RPMI medium supplemented with 2 mM L-glutamine, 1% penicillin/streptomycin, and 10% heat-inactivated FBS. As a negative control, RAW 264.7 cells were cocultured with CM-Dil-labelled MSCs for 12 h, 24 h, and 48 h. The slides were visualized using a Nikon Eclipse Ti-U fluorescence microscope equipped with a digital camera (DS-Ri1, Nikon, Japan).

### Cell vitality

MSCs in good logarithmic growth phase were inoculated in 96-well plates at a density of 5000 cells/well in 100 μL. The control group and SPION group were set up with 5 replicates in each group. After the cells adhered to the wall overnight, LPS was added to stimulate the cells for 24 h, and fresh culture medium added after 24 h. Then, 10 μL of CCK-8 solution was added to each well and incubated for 1.5 h, and the optical density was detected at 450 nm by a microplate reader.

### Cell apoptosis

MSCs were treated with 200 μg/mL SPIONs for 24 h, 48 h, and 72 h, washed once with PBS, and centrifuged at 1300 rpm for 5 min. After the supernatant was discarded, 200 μL of binding buffer was added to each tube for resuspension. Then, 1 μL of annexin V-FITC was added to each tube and mixed well. After the samples were incubated away from light at 4 °C for 15 min, 2 μL of propidium iodide (final concentration: 1 μg/mL) was added to each tube and incubated for 5 min at room temperature in the dark. The cells were subsequently used for flow cytometry.

### Cell migration

MSCs were added to the small chamber. The upper chamber had serum-free medium, and the lower chamber had complete culture solution containing 10% FBS. Then, the cells were placed in an incubator for culture, and MSCs were treated with 200 μg/mL SPIONs for 24 h, 48 h, and 72 h. After removal of the culture solution in the upper chamber and three washes with PBS, the upper chamber was gently swabbed with a cotton swab, the water was removed, and the cells were wiped off the inside of the membrane. Then, ammonium oxalate crystal violet was added to the upper chamber, the samples were dyed for 5 min, the dye liquor was recovered, the dye liquor was slowly washed away with running water, and a cotton swab was used in the upper chamber to absorb water. A slide glass was placed on the inverted microscope, the chamber it was placed on it, images were taken and quantification was performed.

### Small interfering RNAs (siRNAs)

SiRNAs targeting TRAF1 and HO-1 were purchased from RiboBio (Guangzhou, China). MSCs were transfected with 100 nM siRNA or nontargeting siRNA controls using Lipofectamine 2000 reagent (Invitrogen) according to the manufacturer’s instructions and then stimulated with SPIONs for 24 h. SiRNA sequences were as follows: nontargeting control siRNA: 5′-ACGUGACACGUUCGGAGAAUU-3′; TRAF1: 5′-AUCCCAUCCAGAGUUGCUUGUGAUC-3′; HO-1: 5′-TGTCATCCAGCTTACATCTCACAC-3′. TRAF1 and HO-1 mRNA and protein expression levels were assessed by qRT-PCR and western blotting, respectively.

### RNA isolation and qRT-PCR

Total RNA was extracted from tissues and cultured cells using TRIzol Reagent (Invitrogen, USA) according to the manufacturer’s instructions. A total of 1 μg RNA was used as a template for single-strand cDNA synthesis. Q-PCR for GAPDH and siRNAs was performed on an ABI Step One Plus Detection System (Applied Biosystems, USA) using SYBR Green dye (Bio–Rad, USA). Reaction conditions were 95 °C for 10 min followed by 40 cycles of 95 °C for 15 s, 60 °C for 30 s, and 72 °C for 30 s. All reactions were run in triplicate. The gene expression levels were normalized to that of GAPDH.

### Western blotting

Tissue or cells were lysed in buffer containing 50 mM Tris–Cl pH 8.0, 150 mM NaCl, 0.02% NaN3, 0.1% SDS, 100 mg/mL phenyl-methylsulfonyl fluoride (PMSF), 1 mg/ml aprotinin, and 1% Triton. Then, 10 μg/mL aprotinin, 10 μg/mL leupeptin, 1 mM dithiothreitol, 1 mM paranitrophenyl phosphate, and 0.1 mM Na_3_VO_4_ were added as protease and phosphatase inhibitors. After centrifugation, protein samples were subjected to 10% sodium dodecyl sulphate (SDS)-polyacrylamide gel electrophoresis (PAGE) and transferred onto polyvinylidene fluoride (PVDF) membranes (Roche, Mannheim, Germany). Membranes were blocked in TBST (1 mM Tris–HCl, pH = 7.4, 150 mM NaCl, 0.05% Tween-20) containing 5% bovine serum albumin (BSA) for 1.5 h and subsequently incubated overnight at 4 °C with diluted primary antibodies against proteins of interest. All antibodies were used at a dilution of 1:1,000 unless otherwise specified.

### Flow cytometry analysis

For flow cytometry analysis of tissues, liver cells were obtained based on methods described by Nemeth et al. Briefly, freshly removed liver tissues were minced into small pieces and incubated in RPMI 1640 medium with 300 U/mL collagenase type I (Sigma), 300 U/mL collagenase type IV (Sigma), and 50 U/mL DNase I (Sigma). After incubation, cell suspensions were filtered through a 70-µm cell strainer and then washed with complete RPMI medium. Single-cell suspensions were incubated with an Fc receptor blocker (CD16/32, eBioscience) to reduce nonspecific antibody binding. The panel of antibodies used in these experiments included CD11b-APC, F4/80-FITC, CD206 PE, and iNOS-PE (all from eBioscience). Among these antibodies, CD11b and F4/80 cause cell surface staining, while CD206 and iNOS result in both cell surface and intracellular staining. Cells were then washed and stained with iNOS-PE (eBioscience) and CD206-APC (eBioscience). Flow cytometry was performed using a FACSAria flow cytometer (BD Bioscience), and data were analysed with FlowJo software (TreeStar, Ashland, OR).

### DAB prussian blue

The slices were sequentially placed in xylene I for 20 min, xylene II for 20 min, anhydrous ethanol I for 5 min, anhydrous ethanol II for 5 min, and 75% alcohol for 5 min, washed with tap water, and distilled 3 times. Then, the Prussian blue DAB dye solution and Prussian blue DAB dye solution b were mixed in equal proportions. The slices were placed in the mixed solution for dyeing for 30 min and washed twice with distilled water. DAB colour developing solution was prepared by mixing DAB dye solution with oxidizing solution in equal proportion. The DAB colour developing solution was dripped on the slices for approximately 5–10 min, and the degree of colour development was controlled under a microscope. Then, the dye solution was poured, and the slices were washed once with 0.01 mol/L PBS solution, distilled and washed three times. The slices were dyed with Prussian Blue DAB dye solution C for 1 min, washed with tap water, differentiated with hydrochloric acid aqueous solution, washed with tap water, returned to blue with ammonia aqueous solution, and finally washed with tap water.

### H&E staining

Fresh liver tissues, lung tissues, and kidney tissues were fixed in 4% PFA (pH = 7.4), gradually dehydrated, embedded in paraffin, cut into 3-μm sections and stained with haematoxylin and eosin (H&E) for light microscopy. Scores were evaluated based on the presence of epithelial hyperplasia, mononuclear infiltrate, and polymorphonuclear infiltrate (0 = none; 1 = mild; 2 = moderate; 3 = severe).

### Immunofluorescence

After injection of SPION-MSCs or MSCs for 2 days or 7 days, liver tissues were collected, and immunofluorescence staining was used to determine the distribution of MSCs and their localization with macrophages. Briefly, 8-mm-thick frozen liver slices were fixed with cold methanol/acetone (1:1) for 10 min at 220 °C. Following three extensive washes with PBS, the samples were blocked with 3% BSA in PBS for 1 h at room temperature and then incubated with anti-F4/80, anti-CD90, anti-iNOS, or anti-CD206 primary antibody (Abcam, ab6640) at a 1:200 dilution for 2 h. After three washes in PBS, the samples were incubated with Alexa Fluor 488-conjugated secondary antibody at a 1:400 dilution for 1.5 h at room temperature in the dark and then stained with DAPI (Bioworld, China). The slides were visualized using a Nikon Eclipse Ti-U fluorescence microscope equipped with a digital camera (DS-Ri1, Nikon, Japan).

### ELISA

The protein levels of IL-6, TNF-α, and IL-10 in mouse serum were detected using the corresponding mouse ELISA kits according to the manufacturer’s instructions (Biolegend, China).

### Statistical analysis

Statistical analysis was performed using Prism 6 (GraphPad Software). Statistical significance was evaluated by unpaired Student’s t-test. Correlation significance was determined using linear regression. Differences with *p* values ≤ 0.05 were considered statistically significant.

## Results

### SPIONs do not affect the basic characteristics of MSCs

To determine the effect of SPIONs on MSCs, we first detected the basic characteristics of MSCs labelled/pretreated with SPIONs (SPION-MSCs) by fulfilling the minimal criteria for defining MSCs [34]. Prussian blue staining revealed abundant blue-stained particles in the cytoplasm around the red-stained nucleus in the SPION-MSCs. Approximately 100% of the cells exhibited strong intracellular accumulation of SPIONs, indicating a high labelling efficiency of SPIONs. The SPION-MSCs also retained their morphology and capacity to adhere to plastic (Additional file 1: Fig. S1A). Both MSCs and SPION-MSCs maintained their surface markers, including positive expression of CD73, CD90, and CD105 and negative expression of CD34 and CD45 (Additional file 1: Fig. S1B). SPIONs significantly promoted the migratory ability and the expression of the migration-related proteins MMP1 and MMP9 in MSCs in vitro (Additional file 1: Fig. S1C). In addition, no significant differences were detected in either cell viability or apoptosis rate between MSCs and SPION-MSCs at 24 h, 48 h, and 72 h (Additional file 1: Fig. S1D, E). These results indicate that SPIONs did not affect the basic characteristics of MSCs, and SPION-MSCs fulfilled the criteria to define MSCs.

### SPIONs are beneficial for the survival of MSCs under inflammatory conditions

After MSCs are transplanted into the body, they will inevitably face a severely stressful environment. To observe the survival of MSCs under inflammatory conditions, we treated MSCs with 100 ng/mL, 1 µg/mL, and 10 µg/mL LPS for 48 h. The CCK-8 assay results showed that LPS stimulation markedly reduced MSC viability in a dose-dependent manner (Additional file 1: Fig. S2A). Thus, we chose 100 ng/mL LPS for the following experiments. The results showed that the activity of MSCs increased significantly at 24 h and began to decrease significantly at 48 h (Additional file 1: Fig. S2A). HO-1 modification of MSCs can enhance their anti-inflammatory and antioxidant properties and optimize their repair effect. The western blotting results showed that LPS significantly inhibited the expression of the HO-1 protein in MSCs, and the ratio of Bcl2/Bax was decreased in a time-dependent manner (Additional file 1: Fig. S2B). To observe the effect of SPIONs on MSC survival, we treated MSCs with 200 µg/mL SPIONs for 24 h, 48 h, and 72 h. The results showed that SPIONs significantly promoted the expression of the HO-1 protein in MSCs in a time-dependent manner (Additional file 1: Fig. S2C). To further verify the role of SPIONs in regulating the activity of MSCs with HO-1, we synthesized an interfering fragment of HO-1 (si-HO-1). Western blotting experiments showed that SPIONs could restore the low expression of HO-1 and the decrease in the Bcl2/Bax ratio in the LPS-induced MSCs, but the expression level of HO-1 and the ratio of Bcl2/Bax after SPION treatment did not increase (Additional file 1: Fig. S2D). The results of CCK-8 assays and flow cytometry also showed that SPIONs could restore the decrease in MSC activity caused by LPS, but the activity level of the MSCs treated with SPIONs did not increase when HO-1 was inhibited by si-HO-1 (Additional file 1: Fig. S2E–G). Moreover, western blotting analysis showed that SPION-MSCs significantly increased the HO-1 protein levels and the ratio of Bcl2/Bax in the liver tissue of the CLP-induced sepsis group at Day 2 and Day 7. In addition, the ratio of Bcl2/Bax in the SPION-MSC group was higher than that in the MSC group (Additional file 1: Fig. S2H). The data indicate that SPIONs promote the expression of HO-1 to regulate the activity of MSCs in an inflammatory environment, suggesting that SPIONs may be beneficial for the survival of MSCs.

### SPION-MSCs possess enhanced therapeutic efficacy against liver injury in both CLP- and LPS-induced mouse models of sepsis

**Fig. 1:**
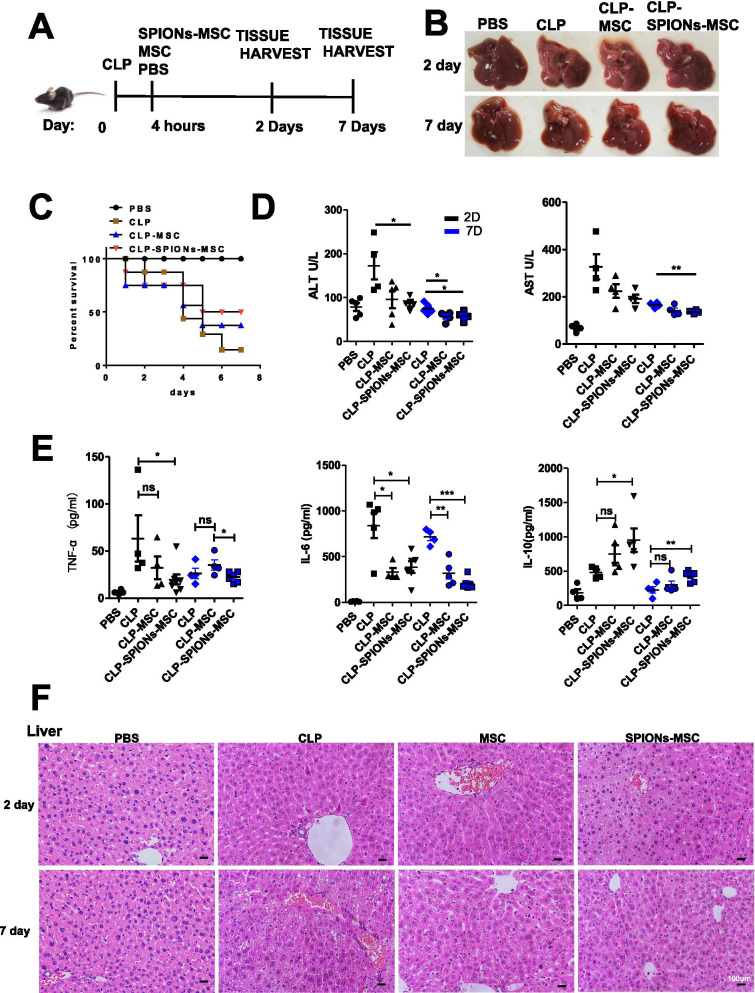
MSCs labelled/pretreated with SPIONs effectively improved sepsis-induced liver injury. **A** Mid-grade sepsis was induced by CLP in male C57BL/6 mice. Sham-operated mice underwent the same procedure without ligation and puncture of the caecum. PBS or a suspension of MSCs or SPIONs-MSCs (10^6^ cells in 150 μl of PBS) was injected via the tail vein 4 h after CLP operation. **B** The mouse liver was photographed to observe its appearance. **C** Survival curves of the mice after CLP and MSC or SPION-MSC therapy. **D** Biochemical indicators of liver function, including serum concentrations of ALT and AST. n = 5–7. **E** Serum concentrations of TNF-α, IL-6 and IL-10 were measured by ELISAs. (F) H&E staining of liver tissues, lung tissues and kidney tissue after sacrifice. Data with error bars are presented as the mean ± SD. Each panel is a representative experiment of at least three independent biological replicates. Scale bars, 50 mm. *p < 0.05, **p < 0.01, ***p < 0.001 as determined by unpaired Student’s t-test

The abovementioned data showed that SPIONs did not affect the basic characteristics of MSCs and were beneficial for the survival of MSCs. Next, we examined the influence of SPION-MSCs on sepsis. The mice with CLP-induced sepsis were randomly grouped and injected with SPION-MSCs, MSCs, or PBS through the tail vein 4 h after a mid-grade CLP operation (Fig. 1A). The visual appearance of freshly dissected liver appeared white and ischaemic in the CLP model mice compared with the normal mice. The results showed that SPION-MSCs recovered more liver colour, improved ischaemia (Fig. 1B) and elevated the survival rate (Fig. 1C) in the mice with CLP-induced sepsis compared with MSCs alone. SPION-MSCs also more significantly decreased the serum levels of liver damage markers such as aspartate aminotransferase (AST) and alanine aminotransferase (ALT) than MSCs (Fig. 1C, D). Moreover, at both Day 2 and Day 7, compared with MSCs, SPION-MSCs significantly decreased the levels of IL-6 and TNF-α but significantly increased the level of the anti-inflammatory factor IL-10 (Fig. 1E). In addition, histological assessment of H&E staining revealed evidence of oedema, inflammatory cell infiltration, and severe haemorrhage in the liver, lung, and kidney from the mice with CLP-induced sepsis (Fig. 1F and Additional file 1: Fig. S3). SPION-MSCs more significantly ameliorated these pathological symptoms than MSCs alone.

To determine the effect of SPION-MSCs on relieving inflammation and regulating macrophages in mice, we also established an LPS-induced sepsis model in mice. The detection indices and methods were the same as those of the CLP-induced mouse model. The results were also similar to those in the CLP-induced mouse model. SPION-MSCs improved the survival rate of the mice (Additional file 1: Fig. S4A), effectively decreased the systemic inflammation level, inhibited the levels of TNF-α and IL-6, and promoted the production of IL-10 (Additional file 1: Fig. S4B, C). SPION-MSCs also alleviated oedema, inflammatory cell infiltration, and severe bleeding in the liver, lung, and kidney of the LPS-induced mice (Additional file 1: Fig. S5A). These data indicate that SPIONs can effectively enhance the therapeutic efficacy of MSCs in both CLP- and LPS-induced septic mouse models and relieve their liver injury.

### SPION-MSC-mediated protection against sepsis-induced liver injury is related to macrophages

**Fig. 2:**
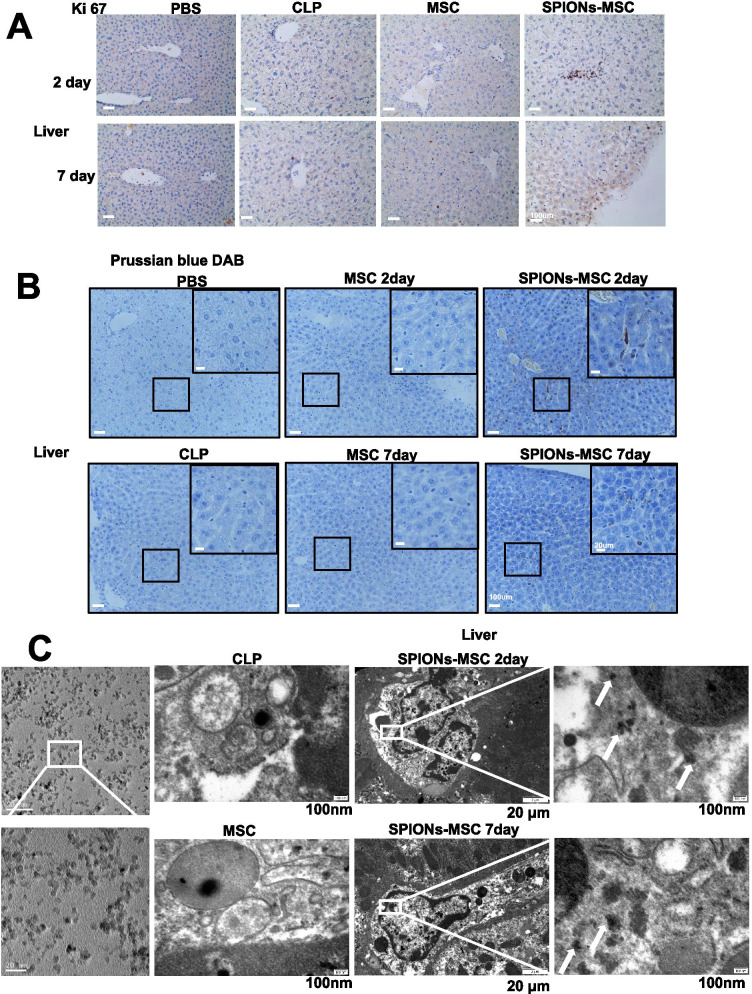
Protection of SPION-MSCs against sepsis-induced liver injury is related to macrophages. **A** Immunohistochemical detection of SPION-MSCs promoted the proliferation of liver cells in septic mice. **B** The livers of mice were stained with DAB Prussian blue. **C** TEM images of macrophages. White arrows show the SPIONs located at the macrophages; control cells without treatment. Data with error bars are presented as the mean ± SD. Each panel is a representative experiment of at least three independent biological replicates. Scale bars, 50 mm. *p < 0.05, **p < 0.01, ***p < 0.001 as determined by unpaired Student’s t-test

To observe the fate and localization of MSCs in the mouse livers, we sacrificed mice 2 days and 7 days after the CLP operation. Ki-67 expression was high in the liver tissue of the CLP-induced sepsis group at Day 2 and Day 7 (Fig. 2A). Then, the liver tissues of the mice were sectioned and stained with DAB Prussian blue. The results showed that brown deposits appeared in the liver on Days 2 and 7 in the SPION-MSC treatment group but not in the other groups (Fig. 2B). TEM was used to analyse macrophages from the livers of different groups of mice, and the results showed that white dense spherical bodies appeared only in the macrophages in the SPION-MSC group (Fig. 2C). These data suggest that macrophages may take up MSC-bearing SPIONs or SPION-MSCs in the liver.

To confirm the correlations of SPION-MSCs and macrophages in the liver, we generated frozen sections and performed double fluorescence staining on liver tissues of different groups of mice. Red-labelled positive liver macrophages (mouse F4/80) were observed under a fluorescence microscope. The results showed that most MSCs labelled with green fluorescence (human CD90 for MSCs) were colocalized with macrophages in the liver tissue of the SPION-MSC treatment groups (Additional file 1: Fig. S6A). These data indicate that macrophages are critical to the enhanced therapeutic efficacy of SPION-MSCs on sepsis-induced liver injury in mice.

### Systemic depletion of macrophages reduces the efficacy of SPION-MSC therapy

**Fig. 3:**
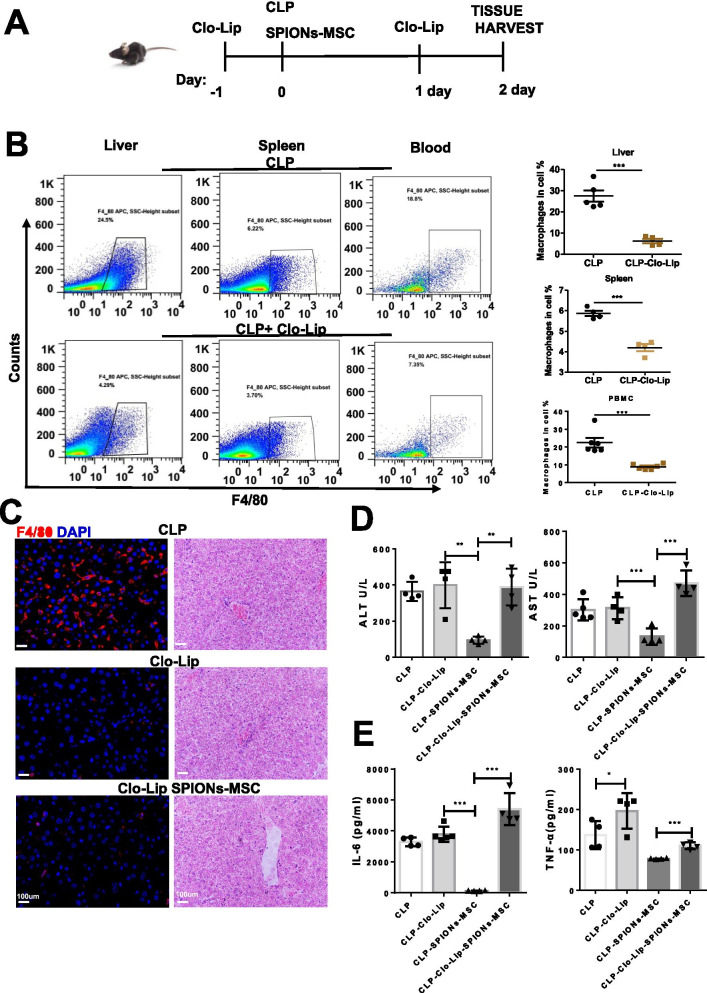
Systemic depletion of macrophages reduces the efficacy of SPION-MSC therapy. **A** Schematic of the macrophage depletion protocol using clodronate liposomes (Clo-Lip). **B** Representative flow cytometry plots of macrophage populations in the spleens, blood, and livers from the Clo-Lip- and PBS-treated animals 2 days after CLP. **C** Immunofluorescence detection and HE pathology of livers from mice. **D** Biochemical indicators of liver function, including serum concentrations of ALT and AST. **E** Serum concentrations of TNF-α and IL-6 were measured by ELISAs. Data with error bars are presented as the mean ± SD. Each panel is a representative experiment of at least three independent biological replicates. Scale bars, 50 mm. *p < 0.05, **p < 0.01, ***p < 0.001 as determined by unpaired Student’s t-test

The transplanted MSCs partially reached the liver, where macrophages absorbed MSCs, and macrophages might play a critical role in the modulation of septic liver injury [35]. To clarify the role of macrophages in the therapeutic effect of SPION-MSCs on sepsis-induced liver injury, we treated the mice with clodronate liposomes to deplete macrophages 24 h prior to CLP (Fig. 3A). As expected, both flow cytometry and immunofluorescence staining showed that the percentage of macrophages was significantly reduced in the liver, spleen, and peripheral blood mononuclear cells (PBMCs) (Fig. 3B). No significant difference was observed in liver function indices between the CLP-induced mice treated with PBS and clodronate liposomes, suggesting that clodronate liposomes themselves did not aggravate sepsis-induced liver injury (Fig. 3C–E). Notably, after depletion of macrophages, SPION-MSCs neither suppressed the serum levels of IL-6 and TNF-α and liver function indicators nor improved the liver structure, as shown by HE staining (Fig. 3C–E). These results indicate that macrophages are indeed necessary for SPION-MSCs to relieve sepsis-induced liver injury in mice.

### SPION-MSCs promote macrophage polarization towards the M2 phenotype under sepsis-induced liver injury in mice

**Fig. 4:**
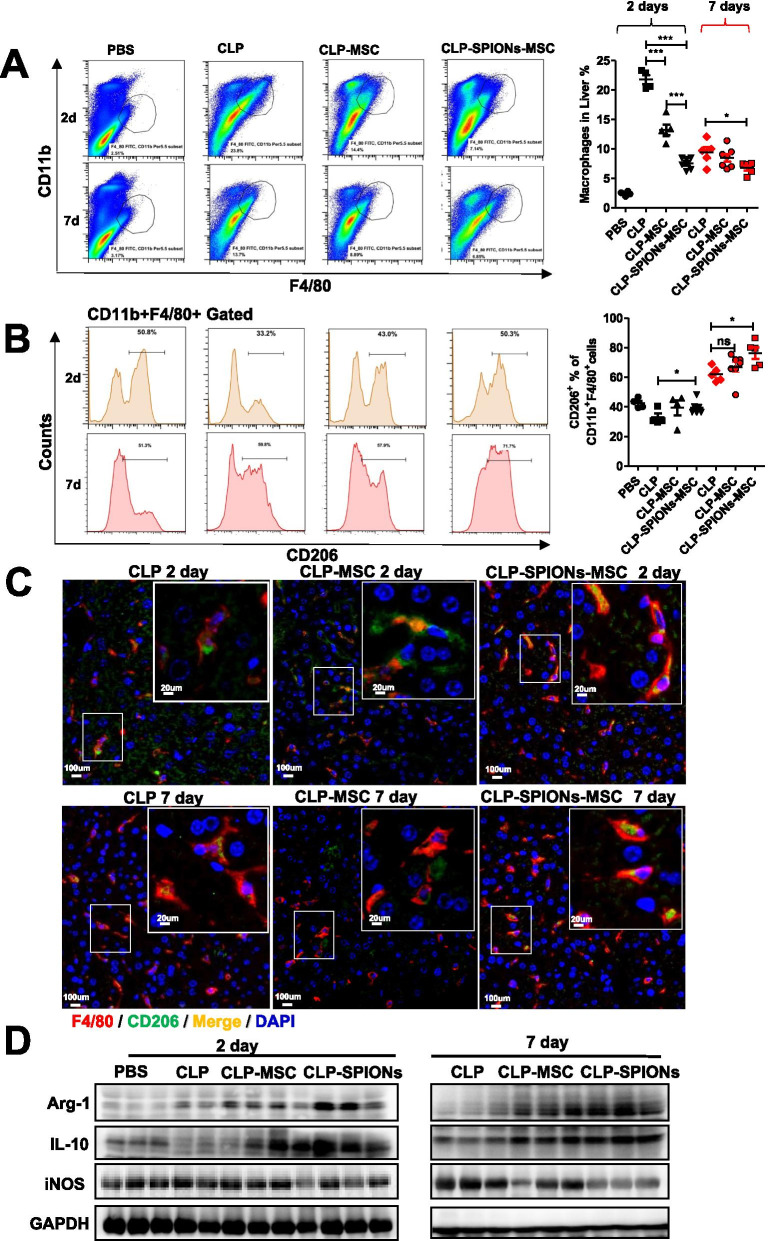
SPION-MSCs promote macrophage polarization towards the M2 phenotype under sepsis-induced liver injury in mice. **A**, **B** Flow cytometry plots and an analysis of the percentage of CD11b + F4/80 + macrophages and the M2 marker CD206. **C** Immunofluorescence of macrophages and their M2 CD206 markers in the whole liver. **D** Representative images of western blots to assess the levels of iNOS, IL-10 and Arg1 in the livers of mice. Data with error bars are presented as the mean ± SD. Each panel is a representative experiment of at least three independent biological replicates. Scale bars, 50 mm. *p < 0.05, **p < 0.01, ***p < 0.001 as determined by unpaired Student’s t-test

To investigate the effects of SPION-MSCs on macrophages, we collected liver tissues at Day 2 or Day 7 from the mice with CLP-induced sepsis in different treatment groups. MSCs, especially SPION-MSCs, significantly decreased the number of macrophages (CD11b^+^F4/80^+^) (Fig. 4A). Macrophage polarization of the M1 type and M2 type generally occurs following injury. We measured the populations of M1 and M2 phenotypes in the mice with CLP-induced sepsis. Flow cytometry analysis showed that, compared with MSC treatment alone, SPION-MSCs remarkably reduced M1 macrophages (CD11b^+^F4/80^+^iNOS^+^) while increasing M2 macrophages (CD11b^+^F4/80^+^CD206^+^) (Additional file 1: Fig. S6A and Fig. 4B). Moreover, we used immunofluorescence staining to analyse the distribution of M1 and M2 macrophages in the liver. The results showed that the colocalization of the red fluorescence of F4/80 and the green fluorescence of CD206 was more significantly increased in the SPION-MSC treatment group than in the MSC treatment group. In contrast, the colocalization of green fluorescence (F4/80) and red fluorescence (iNOS) was more significantly decreased in the SPION-MSC treatment group than in the MSC treatment group (Fig. 4C and Additional file 1: Fig. S7B). Furthermore, western blotting analysis showed that SPION-MSCs significantly reduced the levels of M1 markers, such as iNOS, whereas they increased the levels of M2 markers, such as Arg1 and IL-10 (Fig. 4D). Collectively, these data indicate that SPION-MSCs promote macrophage polarization towards an M2-like state under sepsis-induced liver injury in mice.

### Enhanced polarization towards M2 macrophages is attributed to their phagocytosis of SPION-MSCs

**Fig. 5:**
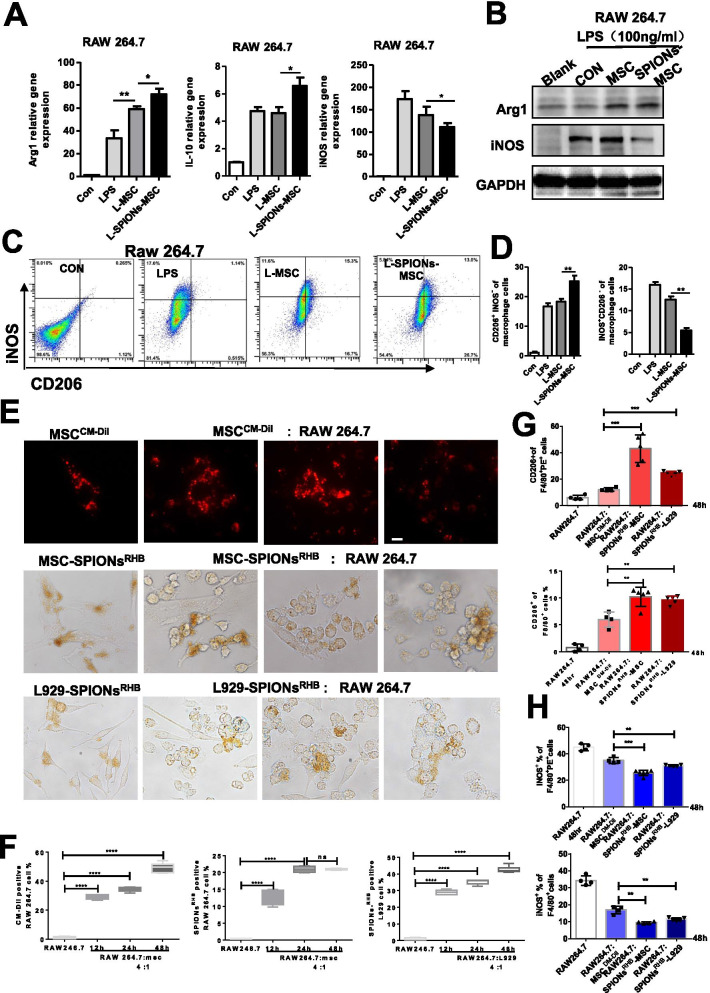
Enhanced polarization towards M2 macrophages is attributed to their phagocytosis of SPION-MSCs. **A** RAW264.7 cells were added to a Transwell system with MSCs or SPION-MSCs for 24 h in the presence of LPS (100 ng/ml). mRNA levels of M1 markers (iNOS) and M2 markers (IL-10 and Arg-1) were detected by qRT-PCR. **B** Protein levels of iNOS and Arg-1 determined by western blots. **C**, **D** Representative flow cytometry plots showing the percentages of M1 (iNOS + CD206 −) and M2 (iNOS − CD206 +) phenotypes in LPS-stimulated peritoneal macrophages after culturing with MSCs or SPION-MSCs for 48 h. **E** RAW264.7 cells with MSCs, SPION-RHB-MSCs or SPION-RHB-L929 cells were directly incubated for 12 h, 24 h or 48 h in the presence of LPS (100 ng/ml). MSCs or L9292 cells: macrophages = 1:4. Representative images of MSCs incubated with CM-Dil, MSCs incubated with SPION-RHB and L929 cells incubated with SPION-RHB. **F** Representative flow cytometry plots showing the percentages of positive cells that engulfed MSCs, SPION-RHB-MSCs or SPION-RHB-L929 cells (F4/80 + PE +). **G**, **H** Engulfing MSCs and percentages of M2 (F4/80 + PE + CD206 −) and M1 ((F4/80 + PE + iNOS +) phenotypes in the LPS-stimulated peritoneal macrophages. Data with error bars are presented as the mean ± SD. Each panel is a representative experiment of at least three independent biological replicates. Scale bars, 50 mm. *p < 0.05, **p < 0.01, ***p < 0.001 as determined by unpaired Student’s t-test

Our above results showed that the regulation of macrophage polarization towards the M2 type by SPIONs-MSCs was necessary to relieve CLP- and LPS-induced sepsis in mice. To further verify the interaction between SPION-MSCs and macrophages under inflammatory conditions, we indirectly cocultured SPION-MSCs with RAW 264.7 cells or BMDMs. We adopted a cell coculture method as shown in Additional file 1: Fig. S8A and coculture in a Transwell system with a 0.4 μm pore size. The results showed that SPION-MSCs more effectively inhibited LPS-induced iNOS production and promoted the upregulation of IL-10 and Arg1 expression in macrophages than MSCs alone (Fig. 5A and Additional file 1: Fig. S8B). Western blotting analysis also showed that SPION-MSCs more dramatically increased Arg1 production in macrophages and lessened the expression of iNOS than MSCs alone (Fig. 5B and Additional file 1: Fig. S8C). Furthermore, flow cytometry showed that SPION-MSCs more significantly increased M2 macrophages but reduced M1 macrophages than MSCs (Fig. 5C, D and Additional file 1: Fig. S8D, E).

We also adopted the method of direct coculture to verify the interaction between SPION-MSCs and macrophages. We bonded SPIONs to the biological dye rhodamine B (RhB) by chemical covalent bonding and then labelled MSCs, which emit red light. Moreover, we used the MSC-labelled living cell tracer CM-Dil (red) and L929 cells labelled with SPION-RhB as a control. RAW 264.7 cells were cocultured with SPION-RhB- or CM-Dil-labelled MSCs or SPION-RhB-labelled L929 cells. The ratio of MSCs or L929 cells to RAW 264.7 cells was 1:4. These cells were observed by microscopy at 12 h, 24 h, and 48 h. The results showed that macrophages began to phagocytose MSCs at 12 h, and most MSCs were phagocytosed by macrophages at 48 h (Fig. 5E). We further detected the proportion of macrophages that phagocytosed MSCs. By flow cytometry analysis, we found that macrophages were increased at 48 h (Fig. 5F and Additional file 1: Fig. S9A). In particular, the proportion of M2 macrophages increased significantly (Fig. 5G and Additional file 1: Fig. S9B), while the proportion of M1 macrophages was reduced in the SPION-RhB-MSC treatment group (Fig. 5H and Additional file 1: Fig. S9C). Similar phenomena were also observed in the SPION-RhB-L929 group. Taken together, these data indicate that the enhanced macrophage polarization towards the M2 type is attributed to the phagocytosis of SPION-MSCs.

### TRAF1 expression in SPION-MSCs is critical for promotion of macrophage polarization and alleviation of sepsis

**Fig. 6:**
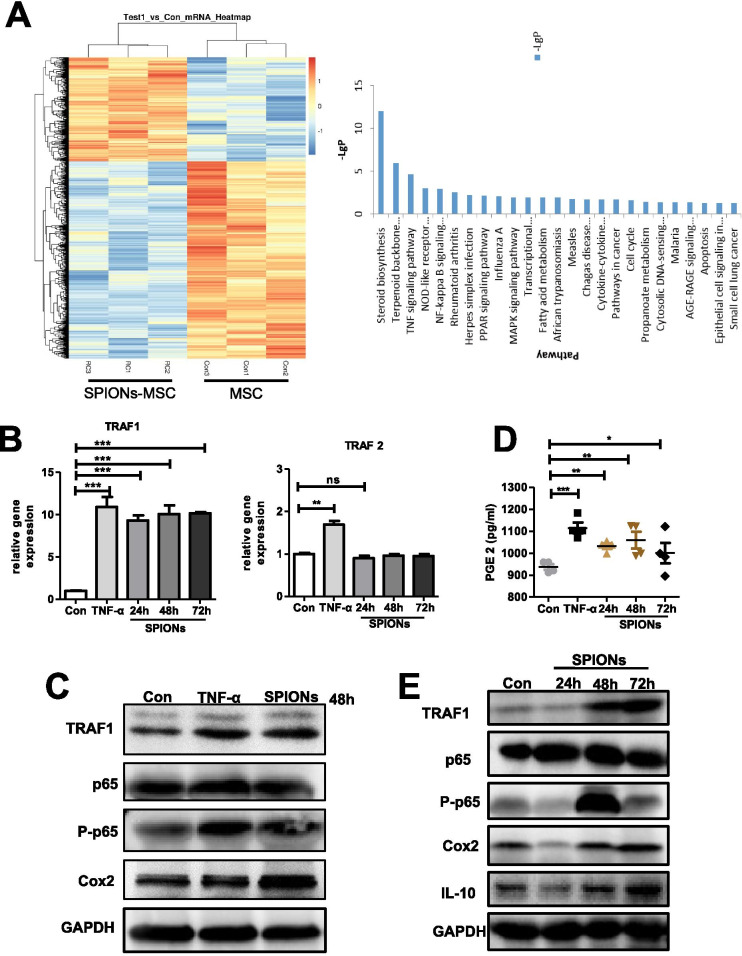
SPIONs promote the high expression of TRAF1 in MSCs to enhance their immune regulation. **A** The thermogram shows that expression of the gene set was significantly upregulated in SPION-MSCs compared with MSCs, and signal pathway enrichment analysis was carried out according to the mRNA sequencing results. **B** mRNA levels of TRAF1 and TRAF2 in the MSCs stimulated with 200 µg/ml SPIONs measured at 24, 48 and 72 h. **C** Protein levels of TRAF1, p65, p-p65, Cox2 and GAPDH in the MSCs stimulated with 200 µg/ml SPIONs or 10 ng/ml TNF-α. **D** PGE2 in culture supernatants was measured by ELISAs. **E** Protein levels of TRAF1, p65, p-p65, Cox2 and GAPDH in MSCs stimulated with 200 µg/ml SPIONs at 24, 48 and 72 h. Data with error bars are presented as the mean ± SD. Each panel is a representative experiment of at least three independent biological replicates. Scale bars, 50 mm. *p < 0.05, **p < 0.01, ***p < 0.001 as determined by unpaired Student’s t-test

To explore the mechanism by which SPION-MSCs enhance macrophage polarization towards M2, we determined the mRNA expression profile of MSCs treated with SPIONs, and MSCs were used as a control group. The whole transcriptome was sequenced by transcriptome sequencing technology, and an algorithm to screen differentially expressed genes was applied. The results showed that SPIONs could activate multiple signal transduction pathways of MSCs, in which 24 genes with high expression were involved in the TNF signalling pathway (Fig. 6A). TNF-α can engage the TNFR-1 receptor to activate the NF-κB pathway and then increase COX2 and PGE2 secretion in MSCs. PGE2 in turn induces macrophages to polarize to the M2 type. For the above reasons, TNF-α-stimulated MSCs were used as a positive control, and 200 μg/mL SPION-treated MSCs were used for 24 h, 48 h, and 72 h. The qRT–PCR results showed that SPIONs induced MSCs to express TRAF1, while the expression of TRAF2 was not affected (Fig. 6B). Furthermore, we detected the concentrations of PGE2 in the culture supernatants and found that SPIONs also induced PGE2. Interestingly, western blotting analysis revealed that TRAF1 levels in MSCs were significantly upregulated in a time-dependent manner. Similar results were also observed for the p65, p-p65, and Cox2 levels (Fig. 6C–E). These data suggest that SPIONs may activate the TNF pathway through TRAF1 to change MSC function, which easily polarizes macrophages towards M2.

**Fig. 7:**
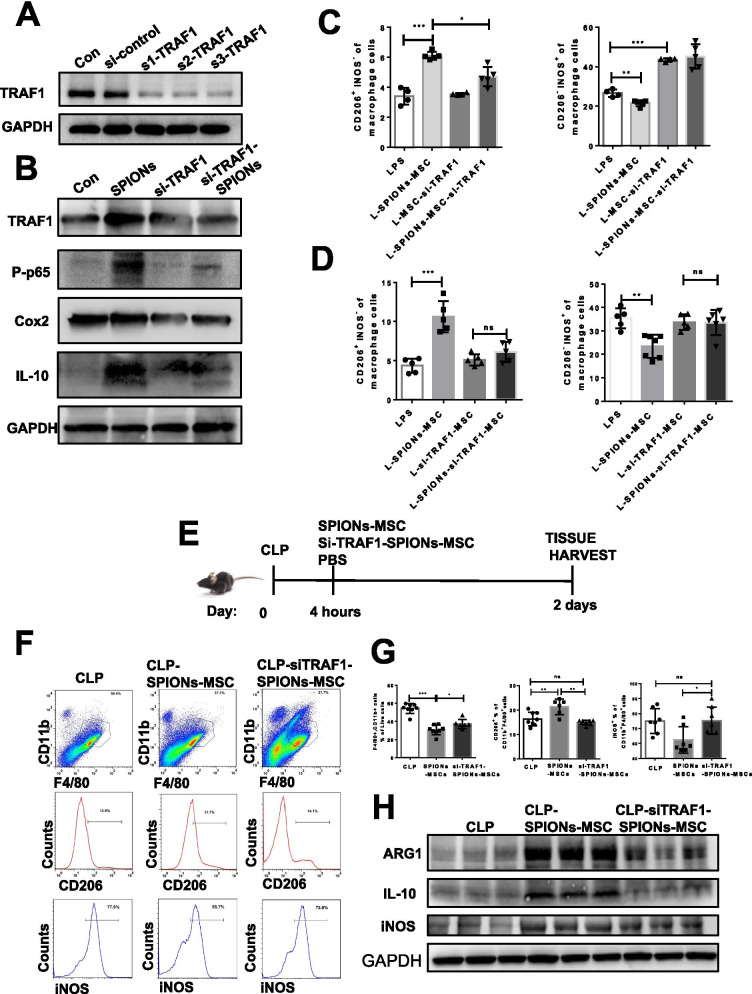
Knockout of TRAF1 weakens the immunomodulatory effect of SPION-labelled/pretreated MSCs on macrophages. **A**, **B** Immunoblotting analysis of TRAF1, p65, p-p65, cox2, IL-10 and GAPDH in the RAW 264.7 cells transfected with si-TRAF1 for 24 h and then stimulated with SPIONs for 24 h. **C**, **D** Representative flow cytometry plots showing the gating strategy used to determine total macrophages, M1 macrophages (iNOS + CD206 −) and M2 macrophages (iNOS-CD206 +). **E** Mid-grade sepsis was induced by CLP in male C57BL/6 mice. Sham-operated mice underwent the same procedure without ligation and puncture of the caecum. PBS or a suspension of SPION-MSCs or siTRAF1-SPION-MSCs (10^6^ cells in 150 μl of PBS). **F**, **G** Flow cytometry plots and analysis of the percentage of CD11b + F4/80 + macrophages and the M2 marker CD206 and M1 marker iNOS. **H** Representative images of western blots to assess the levels of iNOS, IL-10 and Arg1 in the mouse livers. Data with error bars are presented as the mean ± SD. Each panel is a representative experiment of at least three independent biological replicates. Scale bars, 50 mm. *p < 0.05, **p < 0.01, ***p < 0.001 as determined by unpaired Student’s t-test

To verify TRAF1 in the regulatory effect of SPIONs on MSCs, we synthesized an interfering fragment of TRAF1 (si-TRAF1). The protein expression levels of TRAF1, p65, Cox2, and IL-10 and the p65 phosphorylation level were detected by western blotting. The results showed that TRAF1 knockdown significantly inhibited the protein levels of TRAF1, p65, Cox2, and IL-10 and the phosphorylation level of p65 (Fig. 7A, B). SPIONs did not reverse the inhibited TRAF1, P65, Cox2 and IL-10 levels (Fig. 7B).

To determine the role of TRAF1 in SPION-MSCs in polarizing macrophages, we transfected si-TRAF1 or si-control into MSCs. After 24 h, the cells were washed and cocultured with RAW 264.7 cells treated with 100 ng/mL LPS. After another 24 h, the cells stained with CD206 and iNOS antibodies were detected by flow cytometry. The results showed that SPION-MSCs significantly increased M2 macrophages but markedly reduced M1 macrophages compared with those of the MSC group. When si-TRAF1-treated MSCs were cocultured with macrophages in the presence or absence of SPIONs, M1 macrophages were still at a high level, even higher than those in the LPS group (Fig. 7C, D and Additional file 1: Fig. S10A, B).

To further determine whether TRAF1 influences SPION-MSCs to polarize macrophages in the mice with CLP-induced sepsis, we performed experiments as described (Fig. 7E). The results showed that si-TRAF1-SPION-MSCs did not significantly increase M2 macrophages or decrease M1 macrophages (Fig. 7F, G). Moreover, si-TRAF1-SPION-MSCs inhibited the expression levels of Arg1 and IL-10 (Fig. 7H). After knockdown of TRAF1, the effects of SPIONs on MSCs to express cytokines such as Cox2 and IL-10 were also reduced. Furthermore, the immune regulation of MSCs on macrophages was weakened, and the number of macrophages transformed from M1 to M2 was reduced. These results verify that TRAF1 expression in SPION-MSCs is critical for macrophage polarization to M2, the increase in Cox2 and IL-10 levels, and alleviation of sepsis in mice.

## Discussion

Sepsis is now defined as life-threatening organ dysfunction caused by host maladjustment to infection. The liver is the main target organ of sepsis, and sepsis patients will develop severe liver injury and then liver failure. In recent years, MSCs have become an important cell source for alternative treatment of liver diseases. We previously found that SPIONs significantly improved the symptoms of sepsis and liver injury, which was manifested by decreased levels of AST and ALT, decreased TNF-a and IL-6 levels, and increased IL-10 expression. Moreover, stem cell therapy has been used in sepsis, of which development usually leads to multiple organ dysfunction/failure and death [15]. Some studies reported that MSCs derived from bone marrow [36, 37] or adipose tissue [38] were beneficial in mice with dominant sepsis induced by CLP. In addition, human MSCs have been studied in a mouse bacterial pneumonia model [39]. However, it is still unclear how MSCs play roles in immunomodulation and protection of organ injury. Therefore, new strategies are needed to promote MSC function and understand the properties of immune cells in the pathophysiology of sepsis.

In fact, increasing evidence has shown that regardless of intravenous injection or direct local injection, only a very small number of MSCs survive after entering the body [40–42]. Some animal experiments proved that the conditioned medium of MSCs had a therapeutic effect [43, 44]. Therefore, it is speculated that the immunomodulatory function of MSCs might be mainly realized through soluble medium secreted by MSCs or phagocytosis by macrophages. In the present study, MSCs were labelled/pretreated with SPIONs, and the effect of SPION-MSCs on septic liver injury in mice was explored. We found that after MSCs were injected in vivo, they mainly gathered in the liver, contacted macrophages in the liver, or were absorbed by macrophages. The effects of SPION-MSCs on RAW 264.7 cells or BMDMs under direct contact and noncontact conditions were investigated in vitro. These results showed that SPIONs could better promote the transformation of M1 to M2 macrophages under noncontact conditions. In the direct contact experiment, we found that 90% of the macrophages engulfed SPION-MSCs, indicating the M2 type. This finding suggests that SPIONs may enhance the paracrine function of MSCs or influence MSCs themselves. Indeed, both Kupffer cells and monocyte-derived macrophages contribute to the progression and resolution of tissue inflammation and injury in various liver diseases. Thus, it is necessary to clarify the role of KCs and monocyte-derived macrophages in liver damage caused by sepsis in future studies.

Excessive inflammatory reactions lead to the infiltration of macrophages into tissues to cause tissue damage and organ function failure [44, 45]. Currently, macrophages can be categorized into two opposite types: M1 phenotype and M2 phenotype. M1 macrophages are proinflammatory, while M2 macrophages are anti-inflammatory. M2 macrophages can alleviate excessive inflammation and protect tissue damage to a certain extent by secreting anti-inflammatory factors such as IL-10. These cells can also accelerate the clearance of apoptotic cells and participate in tissue repair. Therefore, inhibiting excessive M1 type cells and promoting M2 type polarization in sepsis may be helpful to improve the disease. We established both CLP- and LPS-induced sepsis models in mice and verified that SPION-MSCs significantly improved the symptoms of sepsis, such as increasing the survival rate of mice, reducing the levels of serum TNF-α, IL-6, ALT and AST, and relieving liver tissue damage. Notably, we also found that SPION-MSCs effectively reduced the proportion of M1 macrophages but promoted the proportion of M2 macrophages. Polarization of SPION-MSCs on macrophages may replenish the M2 type in liver tissue.

MSCs have been shown to be effective in treating various inflammatory diseases. Their therapeutic effects require the existence of certain inflammatory conditions. It was reported that IL-1β combined with other inflammatory factors, such as TNF-α, NO, TGF-β, and IFN-γ, enhanced paracrine function and improved the therapeutic effect of MSCs. IL-1β (25 ng/mL) alone also enhanced the migration of MSCs [46, 47]. We previously found that IL-1β-activated MSCs upregulated CXCR4 expression, promoted their migration to inflammatory sites, and improved the symptoms of sepsis in mice [48]. In the present study, we labelled/treated MSCs with SPIONs for 48 h, sequenced their whole transcriptome by transcriptome sequencing technology and applied algorithms to screen differentially expressed genes. We found that SPIONs activated multiple signal transduction pathways in MSCs. Among them, 24 genes were highly expressed and involved in TNF signalling pathways, especially TRAF1. After treatment with 200 μg/mL SPIONs for different times, high TRAF1 gene levels were verified. Moreover, the NF-kB pathway was activated in a time-dependent manner. When TRAF1 was knocked down, we treated MSCs with SPIONs alone for 12 h and then cocultured them with macrophages. The results showed that MSCs' ability to regulate the polarization of macrophages to the M2 type decreased. In contrast, TRAF1 knockdown MSCs were treated with SPIONs (siTRAF1-SPIONs-MSCs) and then injected into CLP-induced septic mice. Their treatment effects were significantly decreased. TRAF1, a member of the TNF receptor family, is an important inflammatory factor. TRAF1 is significantly increased in many inflammatory diseases, such as arthritis, enteritis and sepsis. At present, most studies on TRAF1 focus on its role in the inflammatory environment and the influence of TRAF1 activating the JNK or NF-κB pathway on immune cells. However, TRAF1 has rarely been studied in MSC-based cell therapy. We found that TRAF1 did not affect the basic characteristics of MSCs, including surface markers and cell growth and apoptosis. Therefore, it may be a novel strategy to label/pretreat MSCs with SPIONs to promote the polarization of M2-type macrophages by enhancing the expression of TRAF1 and activating the NF-kb pathway.

In addition, SPIONs could induce MSCs to express HO-1. A certain concentration of LPS promoted a decrease in MSC activity and an increase in apoptosis. LPS also inhibited the expression level of HO-1 in MSCs. We found that SPIONs improved LPS-induced MSC apoptosis related to HO-1 expression. In the liver tissue of the mice with CLP-induced sepsis, we also found that the mice treated with SPION-MSCs had high expression of HO-1 protein and high proliferation of liver cells. However, it is unclear whether HO-1 comes from endogenous MSCs or immune cells of the mouse liver. The specific mechanism and pathway need to be identified.

## Conclusion

In our present study, we found that MSCs labelled/pretreated with SPIONs significantly promoted the polarization of macrophages to the M2 type and improved sepsis and sepsis-induced liver injury in mice. The enhancement of the immunomodulatory function of MSCs labelled/pretreated with SPIONs depends on TRAF1 expression. SPIONs enhance the viability of MSCs by promoting the expression of HO-1. MSCs labelled/pretreated with SPIONs may be a novel therapeutic strategy to prevent or treat sepsis and sepsis-induced liver injury.

## Supplementary Information

**Additional file 1.** Supplementary Figures.

## Data Availability

All data generated or analysed during the study are included in the article.

## Abbreviations

MSCs: Mesenchymal stem cells
SPIONs: Superparamagnetic iron oxide nanoparticles
Arg1: Arginase-1
CLP: Caecal ligation and puncture
Cox2: Cyclooxygenase 2
HO-1: Haem oxygenase 1
TEM: Transmission electron microscopy
LPS: Lipopolysaccharide
PBS: Phosphate-buffered saline
FBS: Foetal bovine serum
AST: Aspartate aminotransferase
ALT: Alanine aminotransferase
IL-6: Interleukin-6
TNF-α: Tumour necrosis factor-α
IL-10: Interleukin-10
BMDMs: Bone marrow-derived macrophages
M-CSF: Macrophage colony-stimulating factor
iNOS: Inducible NOS
PGE2: Prostaglandin E2
TRAF1: TNF receptor-associated factor 1

## References

[1] Kovats S (2011). Climate change, crop yields, and undernutrition, with Sari Kovats by Ashley Ahearn. Environ Health Perspect.

[2] Strnad P, Tacke F, Koch A, Trautwein C (2017). Liver—guardian, modifier and target of sepsis. Nat Rev Gastroenterol Hepatol.

[3] Laroye C, Gibot S, Reppel L, Bensoussan D (2017). Concise review: mesenchymal stromal/stem cells: a new treatment for sepsis and septic shock?. Stem Cells.

[4] Cheng Y, Cao X, Qin L (2020). Mesenchymal stem cell-derived extracellular vesicles: a novel cell-free therapy for sepsis. Front Immunol.

[5] Sriramulu S, Banerjee A, Liddo R, Jothimani G, Gopinath M, Murugesan R (2018). Concise review on clinical applications of conditioned medium derived from human umbilical cord-mesenchymal stem cells (UC-MSCs). Int J Hematol Oncol Stem Cell Res.

[6] Ding L, Yan G, Wang B, Xu L, Gu Y, Ru T (2018). Transplantation of UC-MSCs on collagen scaffold activates follicles in dormant ovaries of POF patients with long history of infertility. Sci China Life Sci.

[7] Atluri S, Manchikanti L, Hirsch J (2020). Expanded Umbilical Cord Mesenchymal stem cells (UC-MSCs) as a therapeutic strategy in managing critically Ill COVID-19 patients: the case for compassionate use. Pain Physician.

[8] Condor J, Rodrigues C, Moreira R, Canale D, Volpini R, Shimizu M (2016). Treatment with human Wharton's Jelly-derived mesenchymal stem cells attenuates sepsis-induced kidney injury, liver injury, and endothelial dysfunction. Stem Cells Transl Med.

[9] Wang Y, Tan L, Jin J, Sun H, Chen Z, Tan X (2015). Non-cultured dermal-derived mesenchymal cells attenuate sepsis induced by cecal ligation and puncture in mice. Sci Rep.

[10] Yuk S, Sanchez-Rodriguez D, Tsifansky M, Yeo Y (2018). Recent advances in nanomedicine for sepsis treatment. Ther Deliv.

[11] Rojas J, Sanz-Ortega L, Mulens-Arias V, Gutierrez L, Perez-Yague S, Barber D (2016). Superparamagnetic iron oxide nanoparticle uptake alters M2 macrophage phenotype, iron metabolism, migration and invasion. Nanomedicine.

[12] Xu Y, Li Y, Liu X, Pan Y, Sun Z, Xue Y (2019). SPIONs enhances IL-10-producing macrophages to relieve sepsis via Cav1-Notch1/HES1-mediated autophagy. Int J Nanomed.

[13] Xu Y, Xue Y, Liu X, Li Y, Liang H, Dou H (2019). Ferumoxytol attenuates the function of MDSCs to ameliorate LPS-induced immunosuppression in sepsis. Nanoscale Res Lett.

[14] Ahrens E, Bulte J (2013). Tracking immune cells in vivo using magnetic resonance imaging. Nat Rev Immunol.

[15] Zheng B, See M, Yu E, Gunel B, Lu K, Vazin T (2016). Quantitative magnetic particle imaging monitors the transplantation, biodistribution, and clearance of stem cells In vivo. Theranostic.

[16] Chen D, Li Q, Meng Z, Guo L, Tang Y, Liu Z (2017). Bright polymer dots tracking stem cell engraftment and migration to injured mouse liver. Theranostics.

[17] Abdollah M, Carter T, Jones C, Kalber T, Rajkumar V, Tolner B (2018). Fucoidan prolongs the circulation time of dextran-coated iron oxide nanoparticles. ACS Nano.

[18] Prockop D (2013). Concise review: two negative feedback loops place mesenchymal stem/stromal cells at the center of early regulators of inflammation. Stem Cells.

[19] Wang L, Li J, Liu H, Li Y, Fu J, Sun Y (2013). Pilot study of umbilical cord-derived mesenchymal stem cell transfusion in patients with primary biliary cirrhosis. J Gastroenterol Hepatol.

[20] Su Z, Li P, Wu B, Ma H, Wang Y, Liu G (2014). PHBVHHx scaffolds loaded with umbilical cord-derived mesenchymal stem cells or hepatocyte-like cells differentiated from these cells for liver tissue engineering. Mater Sci Eng C Mater Biol Appl.

[21] Hu S, Yuan J, Xu J, Li X, Zhang G, Ma Q (2019). TNF-alpha and IFN-gamma synergistically inhibit the repairing ability of mesenchymal stem cells on mice colitis and colon cancer. Am J Transl Res.

[22] Zhao S, Zhang Y, Li M, Zhang X, Chen S (2014). Mesenchymal stem cells with overexpression of midkine enhance cell survival and attenuate cardiac dysfunction in a rat model of myocardial infarction. Stem Cell Res Ther.

[23] Lin C, Wang P, Jin H, Zhao J, Chen D, Liu S (2019). An iron-doped cobalt phosphide nano-electrocatalyst derived from a metal-organic framework for efficient water splitting. Dalton Trans.

[24] Curcio A, Walle A, Serrano A, Preveral S, Pechoux C, Pignol D (2020). Transformation cycle of magnetosomes in human stem cells: from degradation to biosynthesis of magnetic nanoparticles anew. ACS Nano.

[25] Nicolas-Boluda A, Vaquero J, Laurent G, Renault G, Bazzi R, Donnadieu E (2020). Photothermal depletion of cancer-associated fibroblasts normalizes tumor stiffness in desmoplastic cholangiocarcinoma. ACS Nano.

[26] Lartigue L, Wilhelm C, Servais J, Factor C, Dencausse A, Bacri J (2012). Nanomagnetic sensing of blood plasma protein interactions with iron oxide nanoparticles: impact on macrophage uptake. ACS Nano.

[27] Taylor A, Herrmann A, Moss D, See V, Davies K, Williams S (2014). Assessing the efficacy of nano- and micro-sized magnetic particles as contrast agents for MRI cell tracking. PLoS ONE.

[28] Mazuel F, Espinosa A, Luciani N, Reffay M, Borgne R, Motte L (2016). Massive intracellular biodegradation of iron oxide nanoparticles evidenced magnetically at single-endosome and tissue levels. ACS Nano.

[29] Yang G, Gong H, Liu T, Sun X, Cheng L, Liu Z (2015). Two-dimensional magnetic WS2@Fe3O4 nanocomposite with mesoporous silica coating for drug delivery and imaging-guided therapy of cancer. Biomaterials.

[30] Chen L, Zang F, Wu H, Li J, Xie J, Ma M (2018). Using PEGylated magnetic nanoparticles to describe the EPR effect in tumor for predicting therapeutic efficacy of micelle drugs. Nanoscale.

[31] Masthoff M, Buchholz R, Beuker A, Wachsmuth L, Kraupner A, Albers F (2019). Introducing specificity to iron oxide nanoparticle imaging by combining (57)Fe-based MRI and mass spectrometry. Nano Lett.

[32] Xu J, Wang D, Liu D, Fan Z, Zhang H, Liu O (2012). Allogeneic mesenchymal stem cell treatment alleviates experimental and clinical Sjogren syndrome. Blood.

[33] Wang D, Feng X, Lu L, Konkel J, Zhang H, Chen Z (2014). A CD8 T cell/indoleamine 2,3-dioxygenase axis is required for mesenchymal stem cell suppression of human systemic lupus erythematosus. Arthritis Rheumatol.

[34] Dominici M, Blanc K, Mueller I, Slaper-Cortenbach I, Marini F, Krause D (2006). Minimal criteria for defining multipotent mesenchymal stromal cells. The International Society for Cellular Therapy position statement. Cytotherapy.

[35] Li Z, Han S, Jia Y, Yang Y, Han F, Wu G (2019). MCPIP1 regulates RORalpha expression to protect against liver injury induced by lipopolysaccharide via modulation of miR-155. J Cell Physiol.

[36] Capcha J, Rodrigues C, Moreira R, Silveira M, Dourado P, Santos F (2020). Wharton's jelly-derived mesenchymal stem cells attenuate sepsis-induced organ injury partially via cholinergic anti-inflammatory pathway activation. Am J Physiol Regul Integr Comp Physiol.

[37] Laroye C, Boufenzer A, Jolly L, Cunat L, Alauzet C, Merlin J (2019). Bone marrow vs Wharton's jelly mesenchymal stem cells in experimental sepsis: a comparative study. Stem Cell Res Ther.

[38] Silva J, Lopes-Pacheco M, Castro L, Kitoko J, Trivelin S, Amorim N (2019). Eicosapentaenoic acid potentiates the therapeutic effects of adipose tissue-derived mesenchymal stromal cells on lung and distal organ injury in experimental sepsis. Stem Cell Res Ther.

[39] Krasnodembskaya A, Song Y, Fang X, Gupta N, Serikov V, Lee J (2010). Antibacterial effect of human mesenchymal stem cells is mediated in part from secretion of the antimicrobial peptide LL-37. Stem Cells.

[40] Togel F, Hu Z, Weiss K, Isaac J, Lange C, Westenfelde C (2005). Administered mesenchymal stem cells protect against ischemic acute renal failure through differentiation-independent mechanisms. Am J Physiol Renal Physiol.

[41] Lee R, Pulin A, Seo M, Kota D, Ylostalo J, Larson B (2009). Intravenous hMSCs improve myocardial infarction in mice because cells embolized in lung are activated to secrete the anti-inflammatory protein TSG-6. Cell Stem Cell.

[42] Beitnes J, Oie E, Shahdadfar A, Karlsen T, Muller R, Aakhus S (2012). Intramyocardial injections of human mesenchymal stem cells following acute myocardial infarction modulate scar formation and improve left ventricular function. Cell Transplant.

[43] Gupta N, Su X, Popov B, Lee J, Serikov V, Matthay M (2007). Intrapulmonary delivery of bone marrow-derived mesenchymal stem cells improves survival and attenuates endotoxin-induced acute lung injury in mice. J Immunol.

[44] Chen H, Min X, Wang Q, Leung F, Shi L, Zhou Y (2015). Pre-activation of mesenchymal stem cells with TNF-alpha, IL-1beta and nitric oxide enhances its paracrine effects on radiation-induced intestinal injury. Sci Rep.

[45] Kellum J, Kong L, Fink M, Weissfeld L, Yealy D, Pinsky M (2007). Understanding the inflammatory cytokine response in pneumonia and sepsis: results of the genetic and inflammatory markers of sepsis (GenIMS) study. Arch Intern Med.

[46] Munn D, Zhou, Attwood J, Bondarev I, Conway S, Marshall B (1998). Prevention of allogeneic fetal rejection by tryptophan catabolism. Science.

[47] Ge W, Jiang J, Arp J, Liu W, Garcia B, Wang H (2010). Regulatory T-cell generation and kidney allograft tolerance induced by mesenchymal stem cells associated with indoleamine 2,3-dioxygenase expression. Transplantation.

[48] Song Y, Dou H, Li X, Zhao X, Li Y, Liu D (2017). Exosomal miR-146a contributes to the enhanced therapeutic efficacy of interleukin-1beta-primed mesenchymal stem cells against sepsis. Stem Cells.

