# Supplementary material for: SPION-MSCs enhance therapeutic efficacy in sepsis by regulating MSC-expressed TRAF1-dependent macrophage polarization
Source: Stem Cell Res Ther. 2021 Oct 9;12:531. doi: 10.1186/s13287-021-02593-2 (PMC8501658; doi:10.1186/s13287-021-02593-2)
Supplement: Supplementary file 1 — Additional file 1. Supplementary Figures. [file 13287_2021_2593_MOESM1_ESM.pdf]

Graphical Abstract

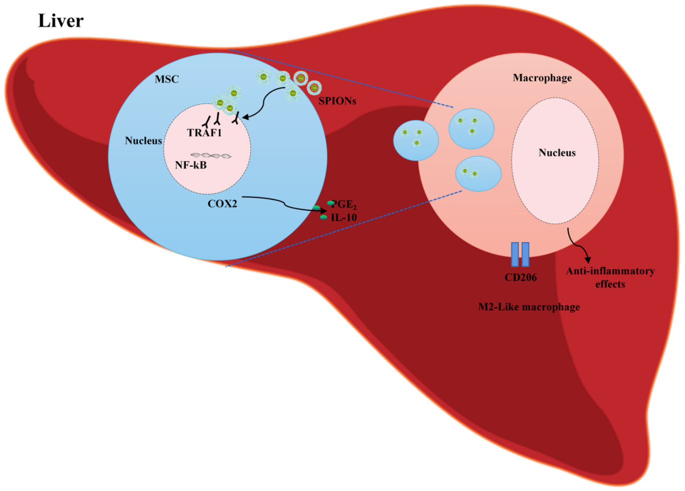

Supplementary figures

Figure S1.

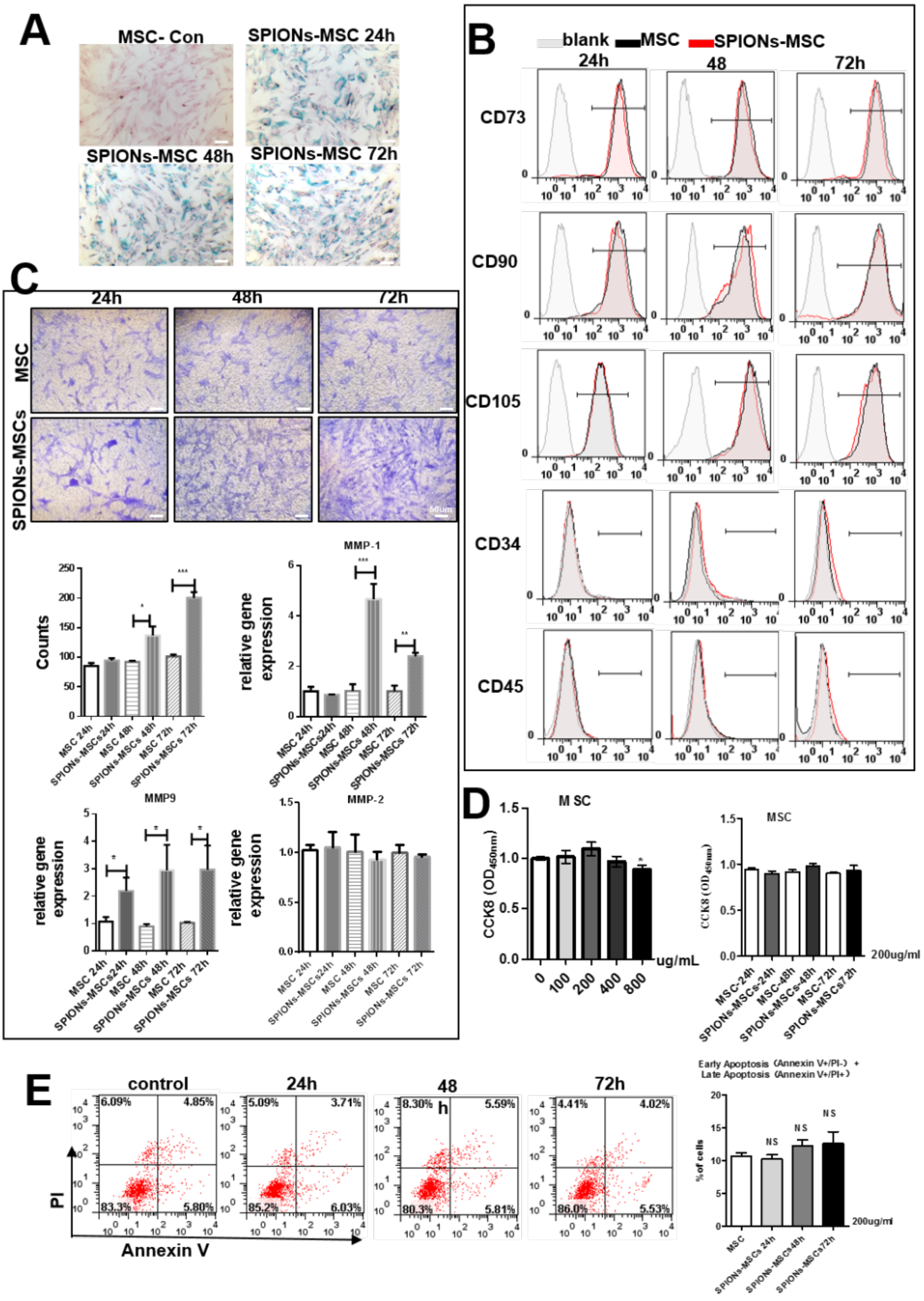

Figure S1. SPIONs do not affect the basic characteristics of MSCs. (A) After MSCs were treated with 200μg/ml SPIONs at 24h, 48h and 72h, Prussian blue staining was performed. (B) Flow cytometric analysis of MSCs surface markers (CD73, CD90, CD105, CD34 and CD45). MSCs, black line; SPIONs-MSCs, red line. (C) The number of MSC migration was observed by crystal violet staining. mRNA expression ofMMP1，MMP2 and MMP9 in MSCs treated with 200 μg/ml SPIONs for 24 h，48h and 72h. (D) MSCs stimulated with 100μg/ml, 200 μg/ml 400μg/ml or 800 μg/ml SPIONs measured at 12, 48, and 72h. (E) Cell viability was determined by CCK8 assay and apoptosis were determined by flow cytomtry. Data with error bars are represented as mean ±SD. Each panel is a representative experiment of at least three independent biological replicates. **p < 0.01 as determined by unpaired Student's t-test. ns, not significant.

Figure S2.

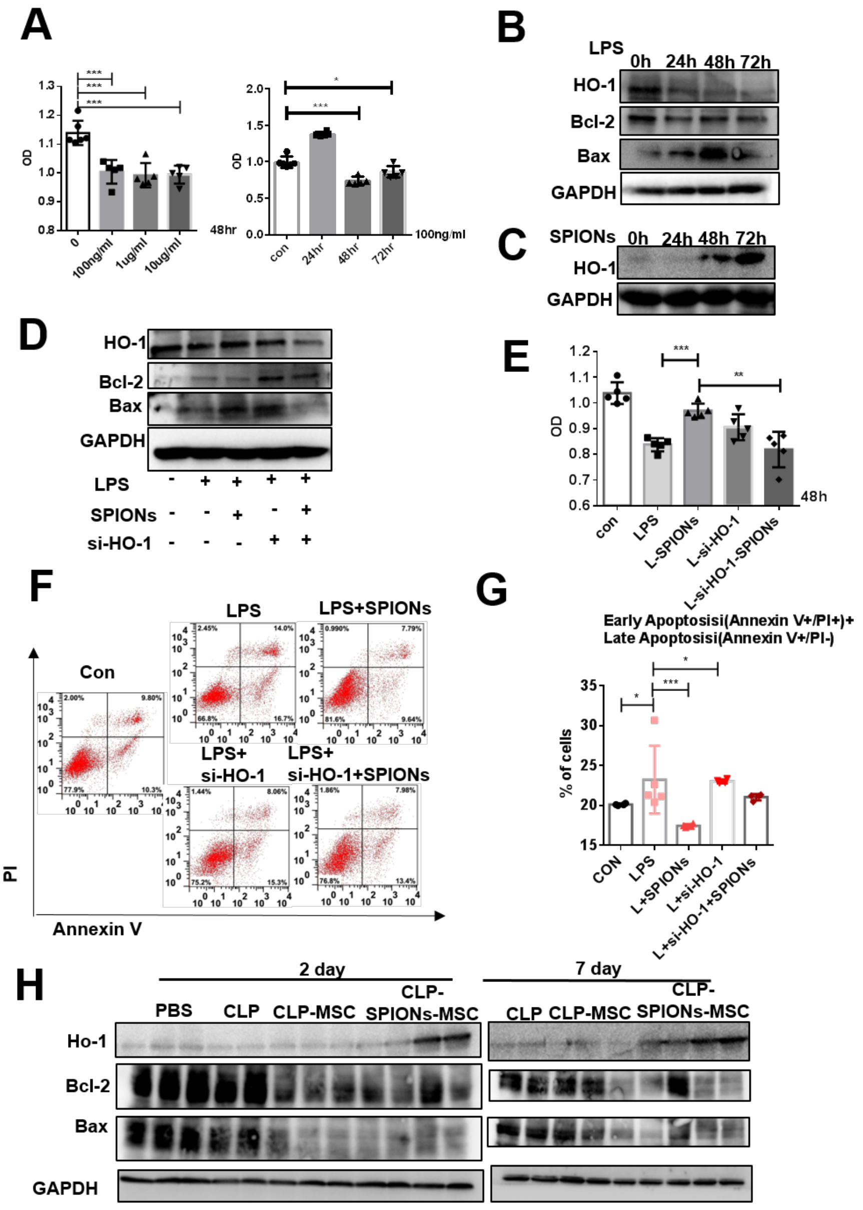

Figure S2. HO-1 plays a key role in SPIONs regulating the growth of MSCs.

(A) CCK8 test of MSCs stimulated with 100ng/ml, 1 µg/ml or 10 µg/ml LPS measured at 24, 48, and 72 h. (B,C) Protein levels of HO-1, Bcl2 and Bax in MSCs stimulated with 100ng/ml LPS or 200ug/ml SPIONs at 24, 48, and 72 h. (D) Immunoblotting analysis of HO-1, Bcl2 and Bax in MSCs transfected with Si-HO-1 for 24 h and then stimulated with SPIONs for 24 h. (E) CCK8 test of MSCs transfected with Si-HO-1 for 24 h and then stimulated with SPIONs or LPs for 48h. (F, G) Apoptosis was determined

by flow cytomtry. (H) Representative images of western blot to assess levels of HO-1, Bcl2 and Bax in the liver of mice. Data with error bars are presented as the mean ± SD. Each panel is a representative experiment of at least three independent biological replicates. Scale bars, 50 mm. *p < 0.05, **p < 0.01, ***p < 0.001 as determined by unpaired Student's t-test.

Figure S3

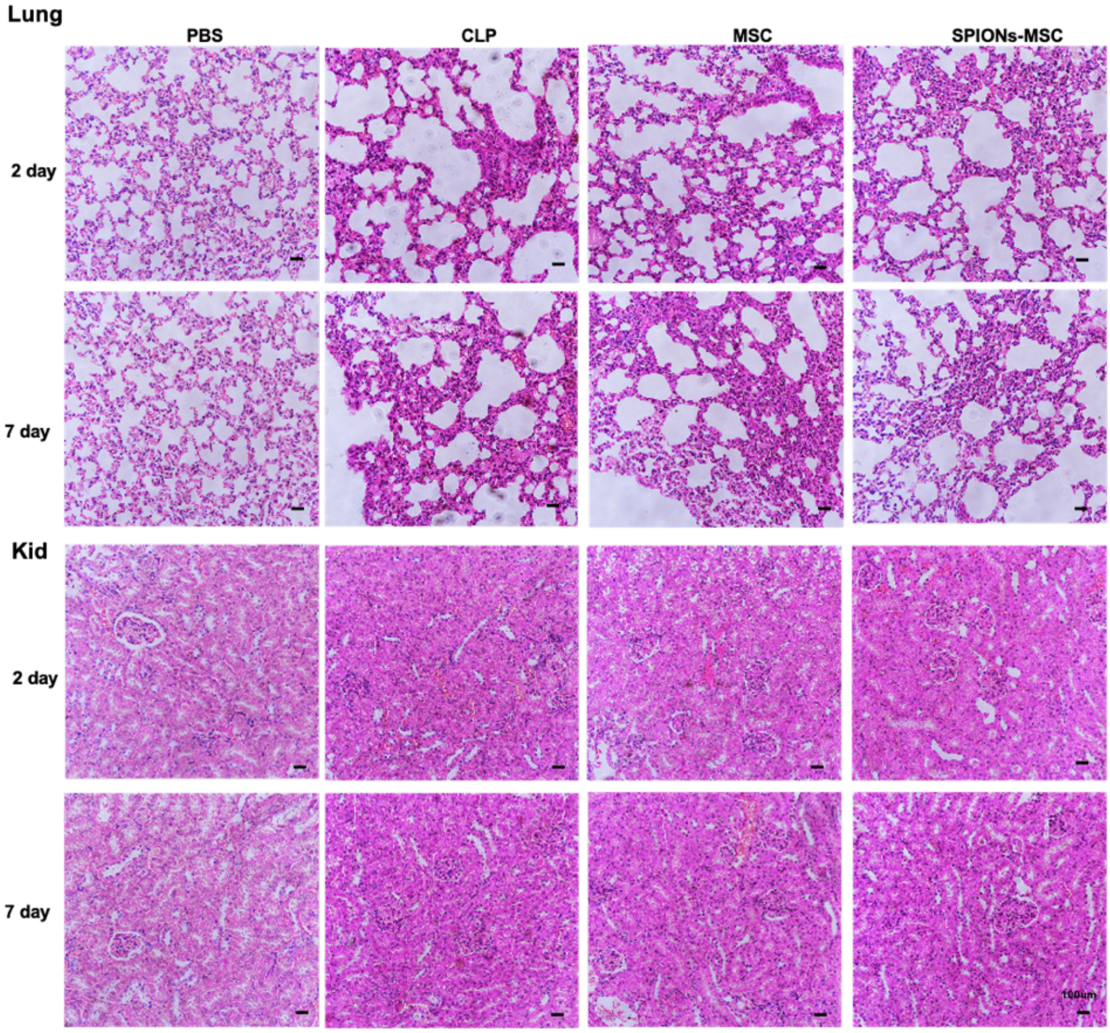

Figure S3. MSCs pretreated with SPIONs effectively improve sepsis-induced liver injury (A) H&E staining of lung tissues and kidney tissues after sacrifice. Data with error bars are represented as mean ± SD. Each panel is a representative experiment of at least three independent biological replicates. *p < 0.05, **p < 0.01, ***p < 0.001 as

determined by unpaired Student's t-test. ns, not significant. Each panel is a representative experiment of at least three independent biological replicates. *p < 0.05, **p < 0.01, ***p < 0.001 as determined by unpaired Student's t-test. ns, not significant.

Figure S4.

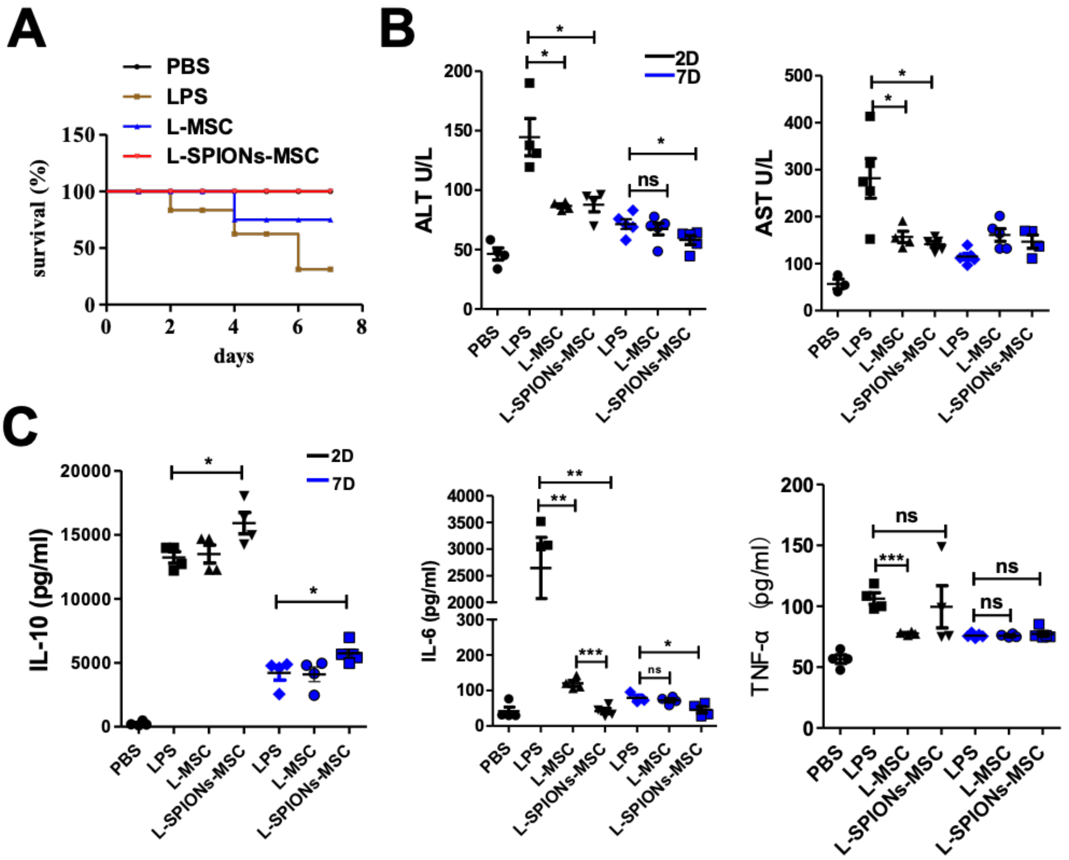

Figure S4. SPIONs-MSCs alleviate the symptoms of liver damage in LPS-induced sepsis mice. (A) Survival curves of mice after LPS and MSCs therapy. (B) Biochemical indicators of liver function including serum concentration of ALT and AST. (C) Serum concentrations of TNF-α, IL-6 and IL-10 were measured by ELISA. Each panel is a representative experiment of at least three independent biological replicates. *p < 0.05, **p < 0.01, ***p < 0.001 as determined by unpaired Student's t-test. ns, not significant.

FigureS5

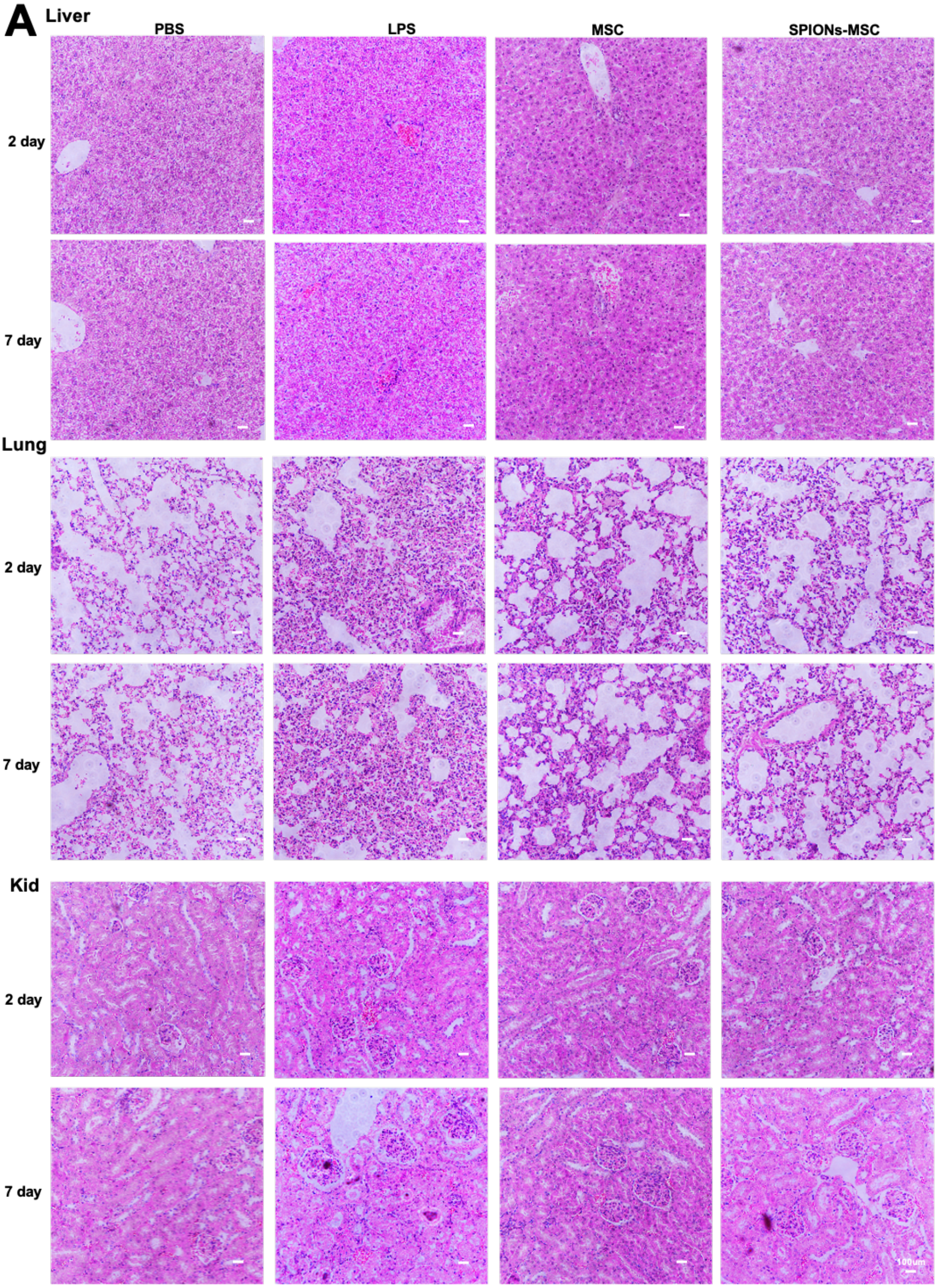

Figure S5. SPIONs-MSCs alleviate the symptoms of liver damage in LPS-induced sepsis mice. (A) H&E staining of liver tissues, lung tissues and kidney tissues after

sacrifice. Data with error bars are represented as mean ± SD. Each panel is a representative experiment of at least three independent biological replicates. *p < 0.05, **p < 0.01, ***p < 0.001 as determined by unpaired Student's t-test. ns, not significant.

Figure S6.

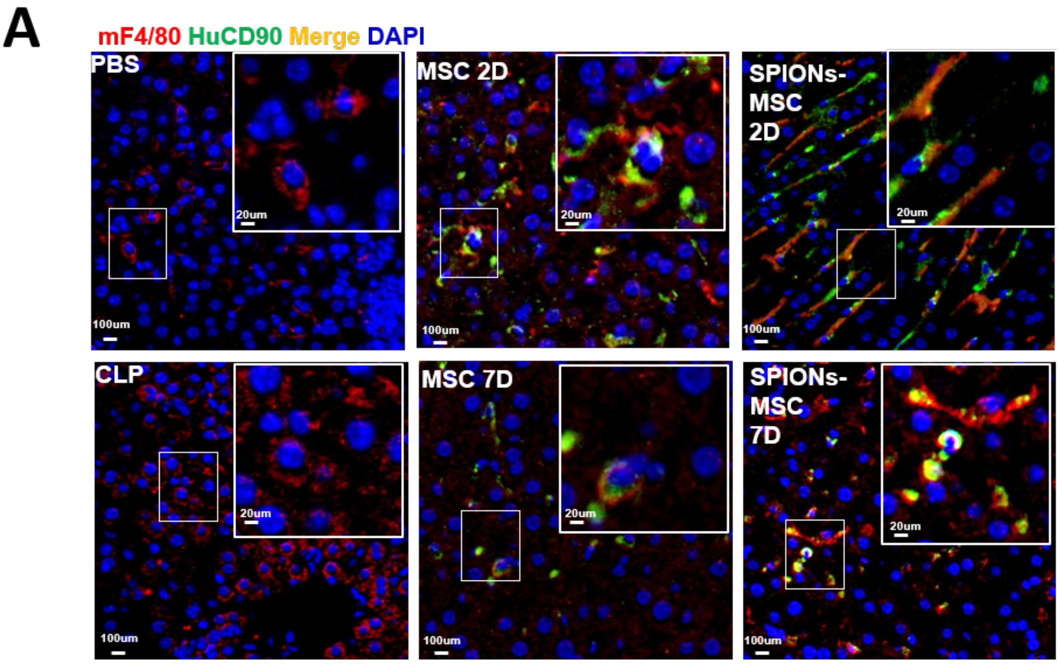

Figure S6. The distribution of MSCs in mouse liver. (A) Confocal immunofluorescent staining of F4/80 (red), Human CD90 (green) and nuclear DAPI (blue) in liver tissue of mice. Data with error bars are represented as mean ± SD. Each panel is a representative experiment of at least three independent biological replicates. ns, not significant.

Figure S7

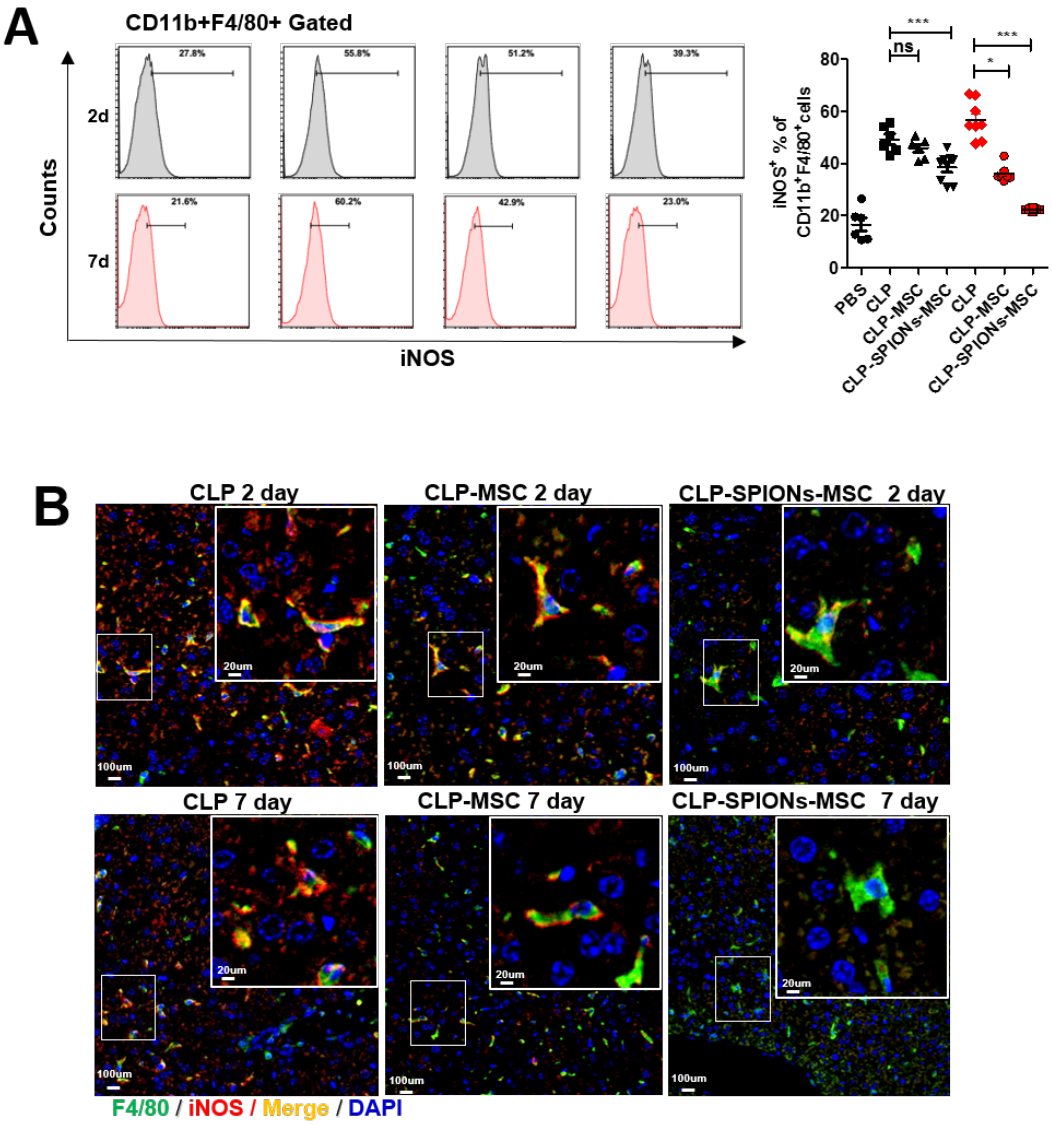

Figure S7. SPIONs-MSCs inhibit macrophage polarization towards M1 type in septic mice. (A) Flow cytometry plots and an analysis of the percentage of CD11b+ F4/80+macrophages M1 markers iNOS. (B) Immunofluorescence of macrophages (F4/80) and their M1 markers (iNOS) in whole liver. Data with error bars are represented as mean ± SD. Each panel is a representative experiment of at least three

independent biological replicates. *p < 0.05, **p < 0.01, ***p < 0.001 as determined by unpaired Student's t-test. ns, not significant.

Figure S8

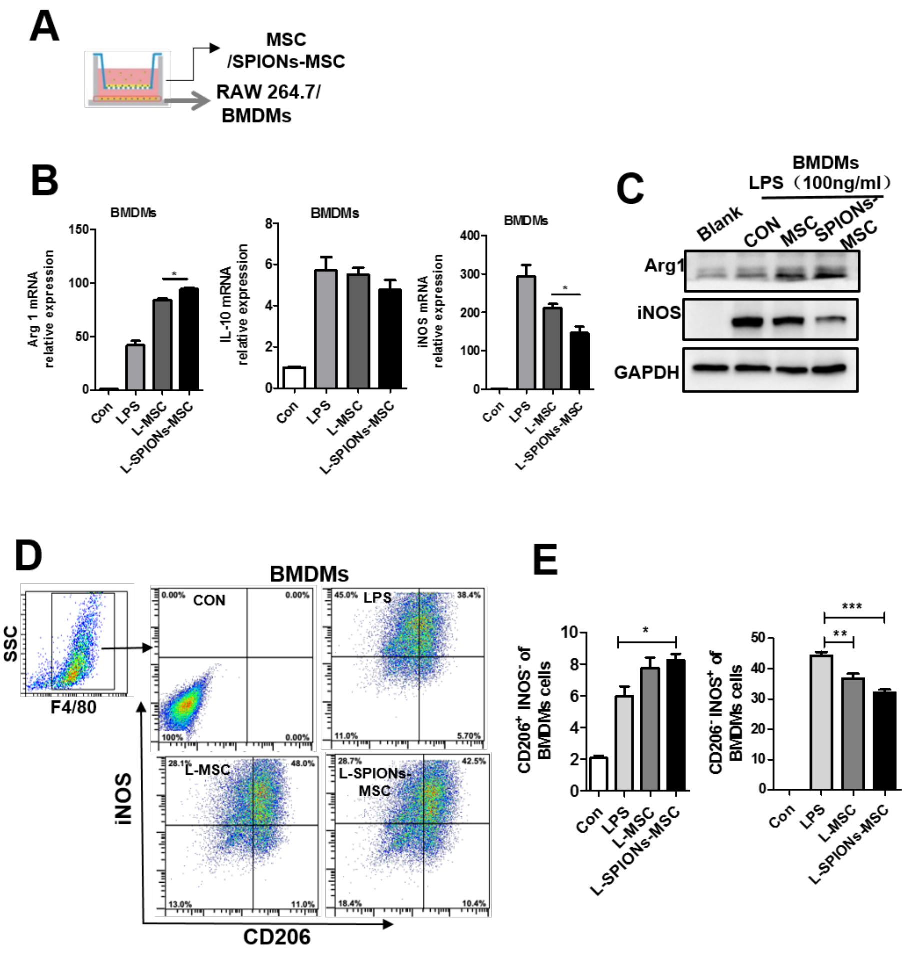

Figure S8. Effects of SPIONs-MSC on the Polarization of BMDMs. (A) BMDMs were cultured with MSCs or SPIONs-MSCs directly or in a for 24 hours in the presence of LPS (100 ng/ml). (B) The mRNA levels of M1 markers (iNOS) and M2 markers (IL-10 and Arg-1) were detected by qRT-PCR. (C) Protein levels of iNOS and Arg-1 were detected by western blot. (D) Representative flow cytometry plots showed the percentages of M1 (iNOS+CD206−) and M2 (iNOS−CD206+) phenotype in LPS-stimulated peritoneal macrophages after culturing with MSC or SPIONs-MSCs for 48 h. Data with error bars are represented as mean ± SD. Each panel is a representative experiment of at least three independent biological replicates. *p < 0.05, **p < 0.01,

***p < 0.001 as determined by unpaired Student's t-test. ns, not significant.

Figure S9

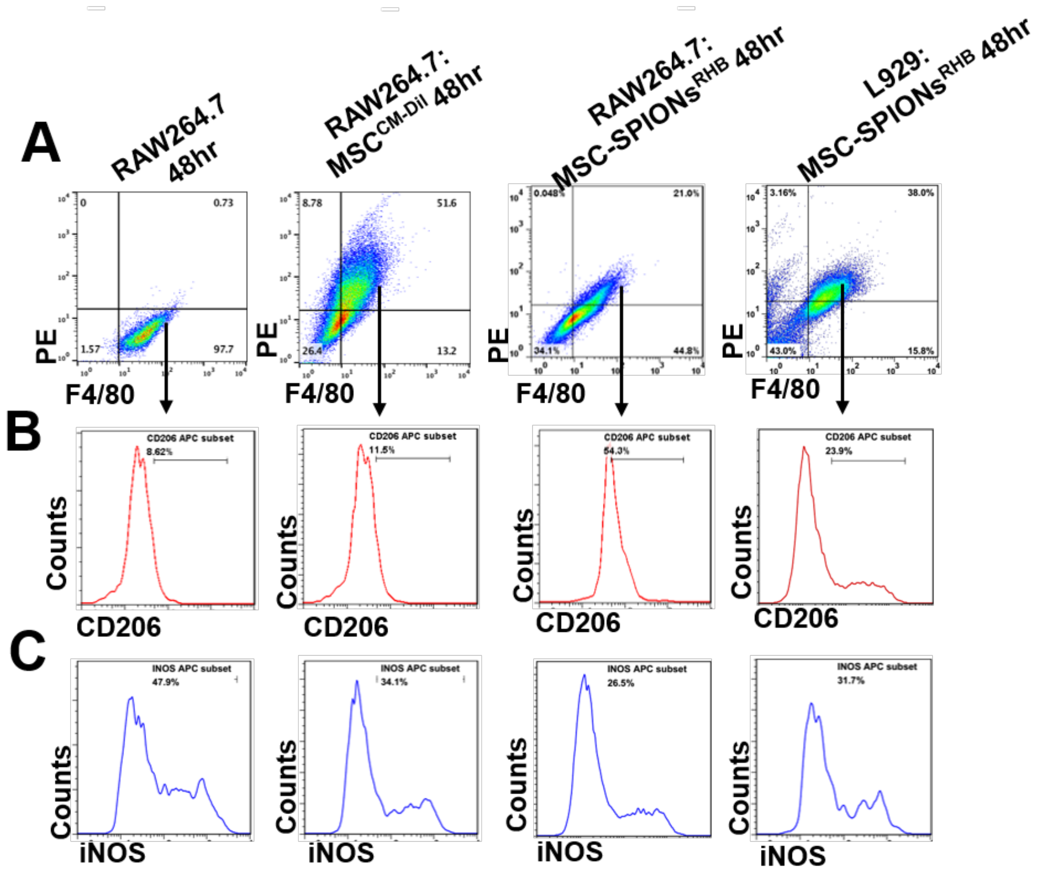

Figure S9. Macrophages phagocytose largely MSCs, SPIONs-RHB-MSCs or SPIONs-RHB-L929. (A-C) Representative flow cytometry plots showed the percentages of M2 (F4/80+PE+iCD206+) and M1 (F4/80+PE+iNOS+) phenotype in LPS-stimulated peritoneal macrophages after culturing with MSCs, SPIONs-RHB-MSCs or SPIONs-RHB-L929 cells for 48 h. Data with error bars are represented as mean ± SD. Each panel is a representative experiment of at least three independent biological replicates. *p < 0.05, **p < 0.01, ***p < 0.001 as determined by unpaired Student's t-test. ns, not significant.

Figure S10

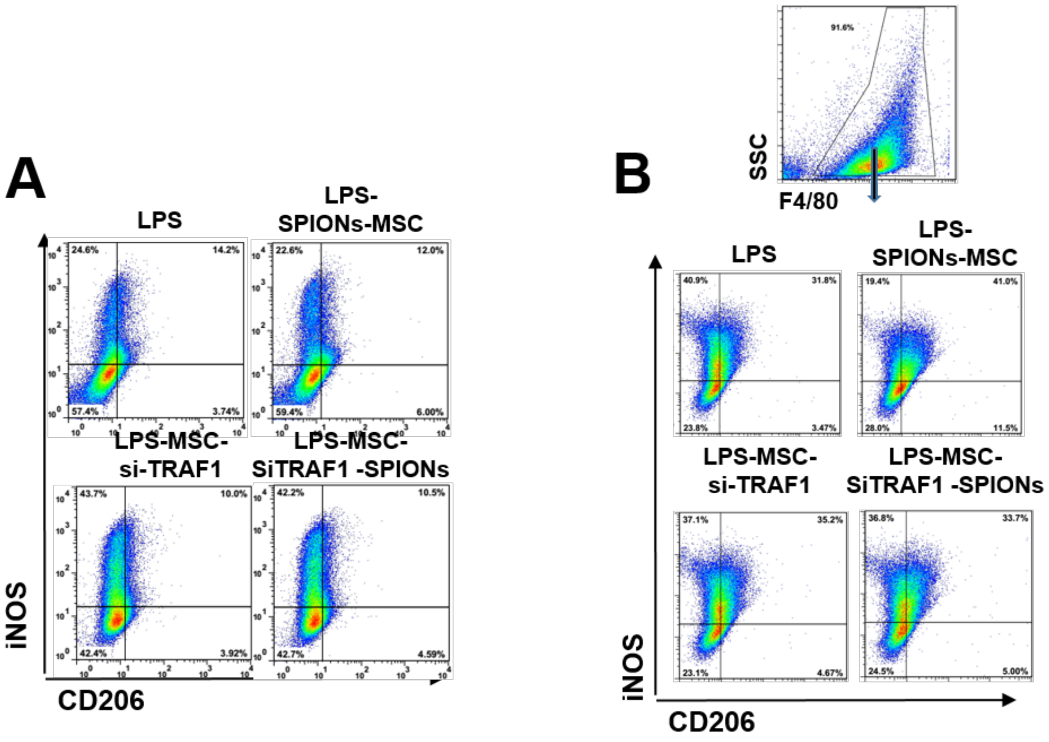

Figure S10. SPIONs promote the high expression of TRAF1 in MSCs to enhance their immune regulation. (A, B) Representative flow cytometry plots showed the gating strategy used to determine total macrophage, M1 phenotype (iNOS+CD206−) and M2 phenotype (iNOS-CD206+). Data with error bars are represented as mean ± SD. Each panel is a representative experiment of at least three independent biological replicates. *p < 0.05, **p < 0.01, ***p < 0.001 as determined by unpaired Student's t-test. ns, not significant.
